# Extreme Energy Spectra of Relativistic Electron Flux in the Outer Radiation Belt

**DOI:** 10.1029/2022JA031038

**Published:** 2022-11-21

**Authors:** D. Mourenas, A. V. Artemyev, X.‐J. Zhang, V. Angelopoulos

**Affiliations:** ^1^ CEA DAM DIF Arpajon France; ^2^ Laboratoire Matière en Conditions Extrêmes CEA Paris‐Saclay University Bruyères‐le‐Châtel France; ^3^ Department of Earth, Planetary, and Space Sciences University of California Los Angeles CA USA; ^4^ Department of Physics University of Texas at Dallas Richardson TX USA

**Keywords:** radiation belt, extreme electron flux, extreme energy spectra, chorus wave, EMIC wave, attractor

## Abstract

Electron diffusion by whistler‐mode chorus waves is one of the key processes controlling the dynamics of relativistic electron fluxes in the Earth's radiation belts. It is responsible for the acceleration of sub‐relativistic electrons injected from the plasma sheet to relativistic energies as well as for their precipitation and loss into the atmosphere. Based on analytical estimates of chorus wave‐driven quasi‐linear electron energy and pitch‐angle diffusion rates, we provide analytical steady‐state solutions to the corresponding Fokker‐Planck equation for the relativistic electron distribution and flux. The impact on these steady‐state solutions of additional electromagnetic ion cyclotron waves, and of ultralow frequency waves are examined. Such steady‐state solutions correspond to hard energy spectra at 1–4 MeV, dangerous for satellite electronics, and represent attractors for the system dynamics in the presence of sufficiently strong driving by continuous injections of 10–300 keV electrons. Therefore, these analytical steady‐state solutions provide a simple means for estimating the most extreme electron energy spectra potentially encountered in the outer radiation belt, despite the great variability of injections and plasma conditions. These analytical steady‐state solutions are compared with numerical simulations based on the full Fokker‐Planck equation and with relativistic electron flux spectra measured by satellites during one extreme event and three strong events of high time‐integrated geomagnetic activity, demonstrating a good agreement.

## Key Points

- Realistic analytical steady‐state electron energy spectra in the presence of wave‐driven energization and loss are provided
- Such steady‐state solutions represent attractors for the system dynamics and correspond to the hardest electron energy spectra
- Steady‐state solutions are compared with numerical simulations and spacecraft observations, showing a good agreement

## 1 Introduction

The observed long‐term dynamics of relativistic electron fluxes in the outer radiation belt has been relatively well reproduced by Fokker‐Planck diffusion codes during various geomagnetic storms (e.g., see Drozdov et al., 2015; Li & Hudson, 2019; Ma et al., 2018; Su et al., 2014; Thorne et al., 2013; Tu et al., 2014). Such Fokker‐Planck codes rely on quasi‐linear diffusion rates for the description of resonant wave‐particle interactions (Andronov & Trakhtengerts, 1964; Kennel & Petschek, 1966; Lyons, 1974). In the inhomogeneous geomagnetic field of the Earth, electron interactions with relatively intense and quasi‐monochromatic chorus whistler‐mode waves can still be accounted for by quasi‐linear theory (Albert, 2010; Karpman, 1974; Karpman & Shkliar, 1977; Tao et al., 2012). Chorus waves consist of intense rising or falling tone elements (Santolík, Gurnett, et al., 2003; Santolík, Parrot, & Lefeuvre, 2003; Tsurutani & Smith, 1974), which are composed of short‐duration wave‐packets/sub‐packets (Santolík, Gurnett, et al., 2003; Santolík, Parrot, & Lefeuvre, 2003; X. J. Zhang, Thorne, et al., 2018) that are often quasi‐monochromatic (R. Chen et al., 2022). Such intense chorus wave‐packets frequently exceed the threshold for nonlinear resonant interaction during substorms (Albert et al., 2013; X. J. Zhang et al., 2019; X. J. Zhang, Thorne, et al., 2018), potentially allowing a much faster electron phase space transport than under the diffusive approximation (O. V. Agapitov et al., 2015b; Allanson et al., 2021; L. Chen et al., 2020; Demekhov et al., 2006; Gan et al., 2022; Miyoshi et al., 2020; Omura et al., 2007; X.‐J. Zhang, Angelopoulos, et al., 2022; X.‐J. Zhang, Artemyev, et al., 2022). However, the prevalence of short chorus wave‐packets and the presence of strong and random wave frequency and phase jumps between and within packets still supports a diffusive description of wave‐particle interactions (Z. An et al., 2022; Artemyev et al., 2021; Mourenas et al., 2018, 2021; Tao et al., 2013; X. J. Zhang et al., 2021; X. J. Zhang, Agapitov, et al., 2020; X. J. Zhang, Mourenas, et al., 2020) where nonlinear contributions may be taken into account via a simple multiplicative factor to diffusion rates of order unity (Artemyev et al., 2021, 2022; Gan et al., 2022; Mourenas, Zhang, et al., 2022).

Both electron inward radial diffusion by ultralow frequency (ULF) waves and chorus wave‐driven electron acceleration are likely contributing to electron flux increases in the outer radiation belt at *L* ≃ 4–6 (Ma et al., 2018; Ozeke et al., 2014, 2020; Thorne et al., 2013). The observed electron flux enhancements often take much more time to develop at higher energies above 1 MeV, which is consistent with a dominant effect of chorus wave‐driven electron energization (Horne et al., 2005; Thorne et al., 2013; X.‐J. Zhang, Mourenas, et al., 2018). In the alternative scenario of a dominant effect of electron inward radial diffusion by ULF waves, although higher energy electrons should then originate from higher *L*‐shells, it should not lead to a similarly significant augmentation of the time delay between electron flux increases at higher and higher energies, due to the fast increase with *L* of the radial diffusion rate *D*_*LL*_ ≈ *L* ^9^ at *L* > 5, corresponding to a faster electron transport at higher *L* (Ozeke et al., 2014). In addition, a peak, and even a growing peak, of 1.5–2.0 MeV electron phase space density (PSD) has been frequently observed at *L* ≃ 4.5–5.5, suggesting a dominant impact of chorus‐wave driven electron acceleration in this region outside the plasmasphere (Allison & Shprits, 2020; Allison et al., 2021; Boyd et al., 2018; Y. Chen et al., 2007; Green & Kivelson, 2004; Tang et al., 2017; Turner et al., 2012, 2013).

In the Earth's outer radiation belt, the great variability of low‐energy (∼10–300 keV) electron injections and betatron acceleration during dipolarization events (Birn et al., 1998, 2012, 2014; Gkioulidou et al., 2015; Liu et al., 2016; Runov et al., 2015; Su et al., 2014; Tang et al., 2022; Turner et al., 2015, 2016), and of the plasma and geomagnetic field conditions determining their subsequent acceleration to higher energy (O. V. Agapitov et al., 2019; Birn et al., 1997; Horne et al., 2005; Summers et al., 1998), are important obstacles to reliable predictions of full relativistic electron flux energy spectra during highly disturbed periods.

Nevertheless, several recent studies have reported the existence of an upper limit on electron fluxes from 300 keV to multi‐MeVs during storm‐time conditions, based on Van Allen Probes observations in 2013–2018 (Hua et al., 2022; Olifer et al., 2021; K. Zhang et al., 2021). This upper limit was found to be roughly inversely proportional to *E* for *E* < 800 keV (Olifer et al., 2021; K. Zhang et al., 2021), apparently consistent with the Kennel‐Petschek theory of electron flux self‐limitation through its generation of whistler mode waves that precipitate electrons into the atmosphere (Kennel & Petschek, 1966; Summers & Shi, 2014). But the Kennel‐Petschek self‐limitation of the electron flux requires sufficiently dense and anisotropic injected hot electron distributions in the considered energy range to generate intense waves, and it further assumes a negligible wave‐driven electron energy diffusion compared to the pitch‐angle diffusion that drives electron loss (Kennel & Petschek, 1966). In the case of chorus waves, such conditions should be satisfied mainly at low energy *E* < 300–500 keV (Horne et al., 2005; Li et al., 2010; Mourenas, Artemyev, Agapitov, & Krasnoselskikh, 2014). At higher energy, chorus wave‐driven electron acceleration can overcome wave‐driven pitch‐angle diffusion loss and rapidly increase the electron flux well above its initial level (Horne et al., 2005; Summers et al., 2002). In such a case, the upper limit on electron flux should be determined in a different way. Using full numerical simulations, Hua et al. (2022) have indeed demonstrated the existence of an upper limit on electron acceleration by chorus waves that can account for the observed saturated electron energy spectrum from ∼0.3–0.5 MeV to ∼2–4 MeV, emphasizing its dependence on electron injections. A full characterization, as a function of all wave and plasma parameters, of the corresponding hardest electron energy spectrum in the outer radiation belt would be useful to define the worst threat to spacecraft electronics (Y. Chen et al., 2021; Hands et al., 2018), but it would require a lot of computer simulations.

As a simpler alternative to full numerical investigations of electron energy spectra, we examine here analytical steady‐state solutions to the Fokker‐Planck equation (Schulz & Lanzerotti, 1974) describing the electron distribution evolution under the influence of strong chorus wave‐driven diffusion, with or without radial diffusion by ULF waves. Such steady‐state solutions should represent attractors for the system dynamics (Lichtenberg & Lieberman, 1983), because the system varies much more slowly in their vicinity. Therefore, such steady‐state analytical solutions are expected to be close to the upper electron energy spectra obtained from full numerical simulations by Hua et al. (2022). In the simplified case of an electron acceleration rate proportional to some power of electron momentum and for an electron loss rate to the atmosphere assumed independent of energy, Bakhareva (2003 2005) was the first to note the existence of such steady‐state solutions to the Fokker‐Planck equation governing electron acceleration by chorus waves and provided the corresponding analytical formulas.

In Section 2, we first examine steady‐state analytical solutions to the Fokker‐Planck equation describing the local dynamics of the relativistic electron distribution, in the presence of chorus wave‐driven electron energization and precipitation into the atmosphere. Recently derived analytical formulations (validated by numerical simulations) of chorus wave‐driven bounce‐averaged quasi‐linear energy and pitch‐angle diffusion rates, and lifetimes, of electrons (Mourenas, Artemyev, Agapitov, & Krasnoselskikh, 2012, 2014; Mourenas, Artemyev, Ripoll, et al., 2012; Mourenas & Ripoll, 2012) are used to provide more realistic steady‐state solutions than in past works (Bakhareva, 2003, 2005; Summers & Stone, 2022), by taking into account the actual dependencies of both electron acceleration and loss rates on energy as well as on wave and plasma parameters. Such analytical steady‐state solutions are compared with numerical solutions. We explore their dependence on various parameters, and their likelihood to be reached within realistic time frames.

Electromagnetic ion cyclotron (EMIC) waves also play an important role in the dynamics of the outer radiation belt, through relativistic electron precipitation into the upper atmosphere (Gao et al., 2015; Ni et al., 2015; Ross et al., 2021; Usanova et al., 2014). Based on Van Allen Probes data, H. Chen et al. (2020) have separately investigated the roles of substorm injection and solar wind pressure in exciting EMIC waves, showing that the source region of EMIC waves driven by substorm injection is located in the dusk sector near the magnetic equator, while solar wind pressure enhancements can cause the excitation of EMIC waves around the noon sector. In Section 3, we provide approximate steady‐state solutions in the additional presence of intense EMIC waves in high‐density plasmaspheric boundary/plume regions at the same *L*‐shell as chorus waves (a situation first investigated numerically by Summers and Ma [2000]), making use of previously derived analytical estimates of the corresponding faster electron loss rates, validated by simulations and observations (Mourenas et al., 2016, 2021; X.‐J. Zhang et al., 2017). In Section 4, we briefly discuss the possible influence of radial diffusion. In Section 5, analytical steady‐state solutions are compared with electron flux observations during periods of high and prolonged geomagnetic activity, most propitious for reaching such stationary states. We show that these steady‐state solutions likely correspond to the hardest electron energy spectra potentially encountered in the outer radiation belt. Therefore, such analytical steady‐state solutions provide a simple means for predicting the most extreme electron energy spectra as a function of all wave and plasma parameters, and geomagnetic activity, in spite of the great variability of magnetospheric conditions.

## 2 Analytical Steady‐State Electron Distribution and Flux Due To Chorus‐Driven Electron Acceleration and Loss

### 2.1 Generalities

In low plasma density regions located outside the plasmasphere, whistler‐mode chorus waves can efficiently stochastically accelerate radiation belt electrons from ∼100–300 keV up to relativistic energies during geomagnetic storms and substorms (O. V. Agapitov et al., 2019; Allison et al., 2021; Horne & Thorne, 1998; Horne et al., 2005; Meredith et al., 2003; Su et al., 2014; Summers et al., 1998; Thorne et al., 2013). Although chorus waves typically consist of series of intense wave‐packets (Santolík, Gurnett, et al., 2003; Santolík, Parrot, & Lefeuvre, 2003) that can reach the threshold for nonlinear wave‐particle interaction (O. V. Agapitov et al., 2015b; Albert et al., 2013; X. J. Zhang et al., 2019), the prevalence of short packets with strong and random wave phase jumps between (and within) packets/subpackets (X. J. Zhang, Agapitov, et al., 2020; X. J. Zhang, Mourenas, et al., 2020), as well as possible interference from other waves (Artemyev et al., 2015), should lead in general to a diffusive chorus wave‐driven evolution of the electron distribution over hours to days (Allanson et al., 2020, 2021; Z. An et al., 2022; Artemyev et al., 2021; Gan et al., 2022; Mourenas et al., 2018, 2021; X. J. Zhang, Agapitov, et al., 2020), which can be approximately modeled by the quasi‐linear diffusion theory (Glauert et al., 2018; Kennel & Petschek, 1966; Mourenas, Zhang, et al., 2022; Thorne et al., 2013).

In the following, we examine this evolution of the electron distribution function $F\left(E,\alpha \right)=A\left(E\right)f\left(p\right)/c^{3}=\left(E+1/2\right)J\left(E,\alpha \right)/\left[c{\left(\left(E+1\right)E\right)}^{1/2}\right]$ (Horne et al., 2005) at *L* = 4.5–6.5, with *f*(*p*) the electron phase space density (where *p* is the electron momentum), *J* the electron differential flux, *A*(*E*) ≃ ((*E* + 1)*E*)^1/2^(*E* + 1/2), assuming equatorial electron pitch‐angles *α* > 50° for the main electron population (Mourenas, Artemyev, Agapitov, Krasnoselskikh, & Li, 2014; Thorne et al., 2013), and where *E* is henceforth in MeV. For an electron flux initially (at *t* = 0) mainly present at low energy (as after a dropout during storm main phase, see Turner et al., 2013) and later evolving self‐consistently under the sole influence of whistler‐mode chorus wave‐electron interactions, the Fokker‐Planck equation describing the evolution of the distribution function *F*(*E*, *α* > 50°) can be written as (Horne et al., 2005):

(1) $$\frac{\partial F}{\partial t}=\frac{\partial }{\partial E}\left[A\left(E\right)D_{EE}\frac{\partial }{\partial E}\left(\frac{F}{A\left(E\right)}\right)\right]-\frac{F}{\tau_{L}}.$$

In Equation 1, the electron lifetime *τ*_*L*_ is the timescale of electron loss into the atmosphere through quasi‐linear pitch‐angle diffusion by chorus waves toward the loss‐cone and *D*_*EE*_ is the chorus wave‐driven bounce‐averaged and MLT‐averaged electron quasi‐linear energy diffusion rate (Horne et al., 2005). Mixed (energy and pitch‐angle) diffusion, which can sometimes have important effects on the evolution of the electron flux (Albert, 2009), has been neglected in Equation 1 to obtain an analytically tractable equation. Mixed diffusion effects are usually weaker for realistically wide statistical distributions of chorus wave‐normal angles and frequencies than for individual narrow‐band waves (Albert, 2009). In a full numerical simulation with realistic chorus wave‐normal angle and frequency distributions (O. V. Agapitov et al., 2018; Horne et al., 2005), the effects of mixed diffusion have been found to remain weak for the high pitch‐angle electrons with *α* ∼ 70° considered here (Albert & Young, 2005).

We assume an initially cold distribution *F*(*E*, *t* = 0) injected at *t* = 0 and examine the self‐consistent evolution of *F*(*E*, *t*) under the sole influence of whistler‐mode chorus waves. Based on previous analytical estimates of *D*_*EE*_, we have $A\left(E\right)D_{EE}=E\left(E+1\right)\left(3/2^{3/2}\right)D_{EE}$ (1 MeV), valid for all *E*, where $D_{EE}$ (1 MeV) depends on wave magnetic power $B_{w}^{2}$ (at the low latitudes of cyclotron resonance with accelerated high *α* electrons), average wave frequency *f*_*m*_ at peak power, electron gyrofrequency *f*_*ce*_, and plasma frequency *f*_*pe*_ (Mourenas, Artemyev, Agapitov & Krasnoselskikh, 2012, 2014). Analytical estimates also show that for *E* > 0.3 MeV and *f*_*pe*_/*f*_*ce*_ ≥ 2, the electron lifetime *τ*_*L*_ can be written as $1/\tau_{L}≃\epsilon \;D_{EE}$ (1 MeV) 3/(2^3/2^[*E* + 1/2][*E*(*E* + 1)]^3/4^), where *ϵ* = 2^5/4^ *E* ^2^/(*D*_*EE*_ *τ*_*L*_) is calculated for *E* = 1 MeV (Aryan et al., 2020; Mourenas, Artemyev, Agapitov & Krasnoselskikh, 2012, 2014). This gives a scaling *τ*_*L*_ *D*_*EE*_/*E* ^2^ ∼ (*E* + 1)^5/4^/*E* ^3/4^. Since analytical estimates of *τ*_*L*_ and *D*_*EE*_/*E* ^2^ include relativistic effects (Mourenas, Artemyev, Agapitov, & Krasnoselskikh, 2012; Mourenas, Artemyev, Ripoll, et al., 2012), the resulting Equation 1 is fully relativistic (Horne et al., 2005). The key factor *ϵ* determines the two possible regimes of electron acceleration: with negligible electron loss for *ϵ* ≪ 1 and with significant loss for *ϵ* ≥ 1 (Mourenas, Artemyev, Agapitov, & Krasnoselskikh, 2014). *ϵ* is independent of energy and mainly depends on the middle latitude to low latitude wave power $B_{w}^{2}$ ratio, corresponding respectively to latitudes of cyclotron resonance with precipitating electrons near the loss‐cone and with high equatorial pitch‐angle electrons accelerated by chorus waves (Horne & Thorne, 2003). *ϵ* also depends on the nightside to dayside plasma density ratio, because electron energization and pitch‐angle diffusion toward the loss‐cone are most important in these respective regions, and plasma density is often lower on the nightside during disturbed periods (O. V. Agapitov et al., 2019; Horne et al., 2005; Mourenas, Artemyev, Agapitov, & Krasnoselskikh, 2014).

Below, we shall mainly focus on analytical steady‐state solutions for the electron distribution, because each of these solutions should represent a kind of attractor for the dynamics of the outer radiation belt, due to the much slower variation of *F*(*E*, *t*) in their vicinity. We shall see that they correspond to particularly hard electron flux energy spectra. Accordingly, these steady‐state solutions provide estimates of the hardest energy spectra that could be encountered during the most extreme geomagnetic events.

### 2.2 Analytical Solutions for *E* > 1.5 MeV

We first examine the high energy part of the distribution at *E* > 1.5 MeV, where we can use the approximations *A*(*E*) ∼ *E* ^2^ and $A\left(E\right)D_{EE}∼E^{2}\left(3/2^{3/2}\right)D_{EE}$ (1 MeV), yielding an analytical solution to Equation 1 for a constant *τ*_*L*_ and a cold initial distribution (Artemyev, Agapitov, et al., 2013; Bakhareva, 2003, 2005; Balikhin et al., 2012; Mourenas, Artemyev, Agapitov, & Krasnoselskikh, 2014):

(2) $$F\left(E,t\right)≃\frac{E^{2}}{D_{EE}^{3/2}t^{3/2}}\exp\left(-\frac{E^{2}}{4D_{EE}\;t}-\frac{t}{\tau_{L}}\right).$$

Equation 2 shows that in this ideal case, for a Dirac‐like electron injection at *t* = 0 and *E* = 0 followed by a self‐consistent evolution, *F*(*E*, *t*) first increases, reaches a maximum for $t≃t_{\max}\left(E\right)=\left[-3/2+{\left(9/4+E^{2}/\left(D_{EE}\tau_{L}\right)\right)}^{1/2}\right]\tau_{L}/2$, and next decreases like 1/*t* ^3/2^ (Bakhareva, 2005; Mourenas, Artemyev, Agapitov, & Krasnoselskikh, 2014). Since *F*(*E*) ≃ *J*(*E*)/*c* for *E* > 0.5 MeV, the shape of electron distribution *F*(*E*) and flux *J*(*E*) are essentially the same in this high energy range. It corresponds to a progressive temperature/energy broadening of the initial cold electron distribution, leading first to an increase of *F*(*E*, *t*) due to the acceleration of abundant low energy electrons up to *E*. After a while, however, more and more electrons from this energy *E* are in turn accelerated to higher energy or lost via precipitation into the atmosphere. Due to conservation of the total number of electrons in the system, this leads to a decrease of *F*(*E*, *t*) when *t* > *t*_max_(*E*).

However, the solution in Equation 2 is usually not very accurate, because the electron lifetime *τ*_*L*_ cannot be taken as constant, since it varies even faster with *E* than *D*_*EE*_ (Artemyev, Mourenas, et al., 2013; Aryan et al., 2020; Mourenas, Artemyev, Agapitov, & Krasnoselskikh, 2014). To get accurate solutions, we can first consider the simplest situation where *ϵ* ≪ 1, equivalent to *τ*_*L*_ *D*_*EE*_/*E* ^2^ ≫ 1. It corresponds to negligible electron loss through precipitation into the atmosphere during the typical timescale of electron acceleration (Mourenas, Artemyev, Agapitov, & Krasnoselskikh, 2014). Then, the maximum of *F*(*E*, *t*) at each energy is reached at *t*_max_ ≃ *E* ^2^/(6*D*_*EE*_) ≪ *τ*_*L*_, and the approximation 1/*τ*_*L*_ → 0 can be safely used in Equations 1 and 2 as long as *t* ≪ *τ*_*L*_. The condition *ϵ* ≪ 1 may be satisfied during extremely active periods with *AE* > 1,000 nT or *Kp* > 6–7 at *L* = 4–6.5 (O. V. Agapitov et al., 2018, 2019), at least when only much weaker EMIC waves are present in high‐density regions on the same *L*‐shells (Mourenas et al., 2016, 2021; X.‐J. Zhang et al., 2017).

In this situation of negligible electron loss, a steady‐state solution satisfying *∂F*/*∂t* = 0 to the Fokker‐Planck Equation 1 with 1/*τ*_*L*_ → 0 must be a solution of the equation

(3) $$\frac{\partial^{2}F\left(E\right)}{\partial E^{2}}-\frac{2}{E}\frac{\partial F\left(E\right)}{\partial E}+\frac{2}{E^{2}}F\left(E\right)=0.$$

The general solution to the Sturm‐Liouville Equation 3 is simply

(4) $$F\left(E\right)=a\cdot E+b\cdot E^{2}$$

with *a* and *b* two constants.

Since physical solutions correspond to *F*(*E*) > 0 at all *E* > 1.5 MeV, the constant *b* must be null or positive and the constant *a* should be such that *a* ≥ 0, or |*a*| < |*b*| if *a* < 0. Consequently, the time‐asymptotic steady‐state *F*(*E*, *t*) and *J*(*E*, *t*) should increase at least linearly with *E* at *E* > 1.5 MeV. Since electron diffusion only acts to reduce gradients in phase space density *f*(*p*) = *c* ^3^ *F*(*E*)/*A*(*E*) ∼ *c* ^3^ *F*(*E*)/*E* ^2^, the fastest possible increase of *F*(*E*, *t*) is like *E* ^2^ at *E* > 1.5 MeV, corresponding to the classical stationary solution with a null gradient *∂f*(*p*)/*∂p* = 0 of electron PSD (Walt, 1994). However, the steady‐state solution in Equation 4 with *F*(*E*, *t*) ∼ *E* (corresponding to *b* = 0) is more likely to be reached asymptotically in time, because it requires much less strong electron injections.

But can the electron distribution *F*(*E*, *t*) reach such asymptotic steady‐state shapes as in Equation 4 in the real magnetosphere? Strong and prolonged injections of low energy electrons from the plasma sheet can continuously bring more low energy electrons that are progressively diffused in energy by chorus waves to higher and higher energy (Meredith et al., 2003; Tang et al., 2017), potentially allowing an unlimited increase of *F*(*E*, *t*) as *t* → +*∞* due to an unlimited increase of the total number of electrons in the system. This suggests that an asymptotic steady‐state solution, satisfying *∂F*/*∂t* = 0, could be reached for sufficiently strong and prolonged injections leading to an approximately constant boundary condition *F*(*E*_0_, *t*) = *F*(*E*_0_, *t* = 0) at low energy *E*_0_ ∼ 300 keV. In the presence of sustained injections, the full distribution *F*_*tot*_(*E*, *t*) can be expressed on the basis of the individual solution *F*(*E*, *t*) for one initial injection, as $F_{\mathrm{tot}}\left(E,t\right)=Q\int_{0}^{t}F\left(E,t^{\prime }\right)dt^{\prime }$ with *Q* the electron injection rate at *E* ≤ *E*_min_, leading to analytical solutions for *E* > 1.5 MeV and a constant *τ*_*L*_ independent of energy (Bakhareva, 2003, 2005). In our case, it gives

(5) $$F_{\mathrm{tot}}\left(E,t\right)=E\cdot \left(\frac{\pi^{1/2}Q}{D_{EE}}\right)\left(1-erf\left[\frac{E}{2\sqrt{D_{EE}\;t}}\right]\right).$$

Equation 5 shows that, in principle, the steady‐state shape *F*_*tot*_(*E*, *t*) ∼ *E* given by Equation 3 could be reached over a finite energy domain *E*_min_ < *E* < *E*_max_ in the presence of sustained electron injections lasting at least until $t>3E_{\max\;}^{2}/D_{EE}\left(E_{\max}\right)$, provided that *t* ≪ *τ*_*L*_(*E*_min_). At higher energy *E* > *E*_max_, *F*_*tot*_(*E*, *t*) is still increasing with time. At very high energy such that *E* ^2^/(6*D*_*EE*_) > *t* (corresponding to *t*_max_(*E*) > *t*), the solution in Equation 2 with 1/*τ*_*L*_ = 0 still applies, forming a steeply decreasing shoulder to the electron distribution, because electrons have not been accelerated in significant numbers up to this high energy. In practice, therefore, the electron distribution can reach a steady state only at not‐too‐high energy *E* < *E*_max_.

In the most general situation, especially during moderately disturbed periods, electron loss into the atmosphere is not negligible. For *E* > 1.5 MeV, we can use the approximation 1/*τ*_*L*_ ∼ *ϵ D*_*EE*_(1MeV) (3/2^3/2^)/*E* ^5/2^. The corresponding approximate steady‐state solution to the full Fokker‐Planck Equation 1 must satisfy the equation

(6) $$\frac{\partial^{2}F\left(E\right)}{\partial E^{2}}-\frac{2}{E}\frac{\partial F\left(E\right)}{\partial E}+\left(\frac{2}{E^{2}}-\frac{\epsilon }{E^{5/2}}\right)F\left(E\right)=0.$$

The general solution to Equation 6 is

(7) $$F\left(E\right)=a\cdot \frac{I_{2}\left(4\;\xi^{1/2}\right)}{\xi^{3}}+b\cdot \frac{K_{2}\left(4\;\xi^{1/2}\right)}{\xi^{3}},$$

where *a* and *b* are two constants, *ξ* = *ϵ*/*E* ^1/2^, and *I*_2_ and *K*_2_ are the modified Bessel functions of the first and second kind, respectively.

The two parts of the steady‐state solution in Equation 7 vary, respectively, like ∼*E* to ∼*E* ^3/4^ and like ∼*E* ^2^ to ∼*E* ^1.5^, for *ϵ* ≪ 1 to *ϵ* ∼ 1. Therefore, the analytical steady‐state solutions given by Equation 7 recover steady‐state solutions in Equation 4 in the proper limit *ϵ* = 0 = 1/*τ*_*L*_. Taking into account realistic chorus wave‐driven electron loss modifies the shape *F*(*E*) of the steady‐state solutions compared to the case without electron loss (*ϵ* = 0). The fastest possible increase of *F*(*E*, *t*) is still like *E* ^2^ (with *a* = 0), corresponding to a null gradient of PSD, *∂f*(*p*)/*∂p* = 0. But this would require injections of high energy *E* > 500 keV electrons, which are usually too rare and much too weak at *L* < 6.5 (Tang et al., 2022) to reach such a null PSD gradient. In the outer radiation belt, electron fluxes are initially steeply decreasing toward higher energy before chorus‐driven energization occurs during storm recovery (Murphy et al., 2018). Therefore, the steady‐state solution in Equation 7 with *b* = 0, which corresponds to the lowest steady‐state *F*(*E*) level (with the slowest increase of *F*(*E*) toward higher *E*), is expected to be observed during chorus‐driven electron energization, because it is the first attractor that will be encountered as high‐energy electron fluxes rise from low initial levels.

### 2.3 Analytical Solutions for All *E*

Let us now relax the previous approximation *E* > 1.5 MeV. Using the full analytical formulas for *D*_*EE*_, *A*(*E*), and *τ*_*L*_, the general steady‐state solution to Equation 1, valid for all *E* and *ϵ* values, is given by:

(8) $$\begin{array}{ccc} \left(1+4E+36E^{2}+64E^{3}+32E^{4}-4\;\epsilon \;E^{3/4}{\left(E+1\right)}^{3/4}{\left(2E+1\right)}^{2}\right)\;F+ & & \\ +\;4\;E^{2}{\left(E+1\right)}^{2}{\left(1+2E\right)}^{2}\;\frac{\partial^{2}F}{\partial E^{2}}-16\;E^{2}{\left(E+1\right)}^{2}\left(1+2E\right)\;\frac{\partial F}{\partial E}=0, & & \end{array}$$

First, we consider the case of negligible electron loss, with *ϵ* ∼ 0. In this case, the general Equation 8 has an exact solution

(9) $$F\left(E\right)=\left(a\;\left[\ln\left(E+1\right)-\ln\left(E\right)\right]+b\right)\cdot \left(2E+1\right)\sqrt{E}\sqrt{E+1},$$

where *a* and *b* are two constants. The two different exact steady‐state solutions in Equation 9 vary over *E* = 0.3–1 MeV like ∼*E* ^0.55^ for *b* = 0 and like ∼*E* ^1.2^ for *a* = 0, respectively. Over 1–10 MeV, they vary like ∼*E* ^0.9^ and ∼*E* ^1.8^, respectively, in agreement with the approximate steady‐state solutions in Equation 4 valid for *E* > 1.5 MeV. Since physical solutions must correspond to *F*(*E*) > 0 at all *E* > *E*_min_ ∼ 0.3 MeV, the constants *a* and *b* in Equation 9 must satisfy *b* ≥ 0 and either *a* ≥ 0 or |*a*| < 0.41|*b*| if *a* < 0 and *b* > 0.

The most general steady‐state form of *F*(*E*, *t*), in the presence of sustained injections at low energy *E*_0_ and chorus wave‐driven electron acceleration and precipitation into the atmosphere, is the solution to the full Equation 8. However, this full equation is too complex to get a simple, exact analytical solution valid for all energies and *ϵ* values. Numerical calculations of chorus wave‐driven electron lifetimes and energization rates at *L* = 4.0–6.5 outside the plasmasphere show that *ϵ* ∼ 0.5–0.9 during very active periods with *AE* ∼ 600–800 nT or *Kp* ∼ 5–6 based on chorus wave statistics obtained from combined Van Allen Probes and Cluster spacecraft data (O. V. Agapitov et al., 2018, 2019). Accordingly, we first take *ϵ* ∼ 0.65 as representative of a typical case of high geomagnetic activity with significant electron loss. In such a case, we can use the approximation (1 + 4*E* + 36*E* ^2^ + 64*E* ^3^ + 32*E* ^4^ − 2.6*E* ^3/4^(*E* + 1)^3/4^(2*E* + 1)^2^) ≃ 25*E* ^2^(1 + *E*)^2^, with less than 5% error over ∼0.3–2 MeV, less than 10% error over 2–5 MeV. The full Equation 8 can then be approximated as

(10) $$4{\left(1+2E\right)}^{2}\;\frac{\partial^{2}F\left(E\right)}{\partial E^{2}}-16\left(1+2E\right)\;\frac{\partial F\left(E\right)}{\partial E}+25\;F\left(E\right)=0,$$

with an exact solution

(11) $$F\left(E\right)=a\cdot {\left(2E+1\right)}^{\left(\frac{3}{2}-\sqrt{\frac{11}{16}}\right)}+b\cdot {\left(2E+1\right)}^{\left(\frac{3}{2}+\sqrt{\frac{11}{16}}\right)},$$

where *a* and *b* are two constants. The first and second parts of this approximate solution (Equation 11) vary like *F*(*E*) ∼ *E* ^0.45^ and *F*(*E*) ∼ *E* ^1.4^, respectively, over *E* ∼ 0.3–2 MeV. Comparing steady‐state solutions in Equations 9 and 11 for *b* = 0 shows that including a significant electron loss restrains the increase of *F*(*E*) with *E*. A similar approximation, (1 + 4*E* + 36*E* ^2^ + 64*E* ^3^ + 32*E* ^4^ − 4*ϵ E* ^3/4^(*E* + 1)^3/4^(2*E* + 1)^2^) ≃ Υ *E* ^2^(1 + *E*)^2^, can be used for other *ϵ* values, with less than 10% error over ∼0.3–5 MeV for 0.4 < *ϵ* ≤ 0.85 and less than 20% error over ∼0.3–4 MeV for 0.85 < *ϵ* ≤ 1.0. The corresponding steady‐state solutions have a form (2*E* + 1)^3/2±*g*(*ϵ*)^ as in Equation 11. Further taking into account that we must recover the exact solution in Equation 9 with *b* = 0 for *ϵ* → 0, and searching for a simple analytical best fit to *g*(*ϵ*), finally yields an approximate general steady‐state solution

(12) $$F\left(E\right)=a\cdot {\left(2E+1\right)}^{\left(\frac{3}{2}-{\left[\frac{3}{16}+\frac{10\;\epsilon^{2}}{9}\right]}^{1/2}\right)}+b\cdot {\left(2E+1\right)}^{\left(\frac{3}{2}+{\left[\frac{3}{16}+\frac{10\;\epsilon^{2}}{9}\right]}^{1/2}\right)}$$

valid for 0 ≤ *ϵ* ≤ 1 and 0.3 MeV < *E* < 5 MeV, with *a* and *b* two constants. For *ϵ* = 0.65, Equation 12 with *b* = 0 gives a variation *F*(*E*, *t*) ∼ *E* ^0.63^ over 2–5 MeV, close to the variation *F*(*E*, *t*) ∼ *E* ^0.75^ of the approximate solution in Equation 7 derived by directly taking the limit *E* ≫ 1 MeV in expressions of *D*_*EE*_, *τ*_*L*_, and *A*(*E*).

However, the first term (1 + 4*E* + 36*E* ^2^ + 64*E* ^3^ + 32*E* ^4^ − 4*ϵ E* ^3/4^(*E* + 1)^3/4^(2*E* + 1)^2^) in Equation 8 can become null or negative when *ϵ* > 2^1/2^, with negative values at ∼0.3 MeV for *ϵ* ∼ 1.5 and up to 0.75 MeV for *ϵ* ∼ 2. In such a situation, there is no simple analytical solution valid at all *E*. This situation with *ϵ* > 2^1/2^ corresponds to chorus wave‐driven electron losses faster than electron acceleration at *E* < 1 MeV. Such conditions are encountered during the most common periods with average *AE* < 400 nT and *Kp* < 4 (O. V. Agapitov et al., 2018; Agapitov et al., 2019). In this situation, the steady‐state *F*(*E*) should decrease from ∼0.3 to ∼0.75 MeV, while it should only weakly increase with *E* at higher energy. The approximate solution given by Equation 12 for *b* = 0 indeed shows that *F*(*E*) increases more and more slowly with *E* as *ϵ* increases toward 1.

Equations 9 and 12 provide the full scaling with *E* of the most extreme steady‐state electron distributions with and without electron loss, obtained when *ϵ* ≤ 1. As noted before, in the outer radiation belt, it is the steady‐state solution with *b* = 0, corresponding to the lowest steady‐state *F*(*E*) (increasing the most slowly toward higher *E*), that is expected to be observed during chorus wave‐driven electron energization, because it is the first encountered attractor as high‐energy electron fluxes rise from low initial levels (Mourenas et al., 2019; Murphy et al., 2018). Without electron loss (for *ϵ* = 0), the steady‐state *F*(*E*) with *b* = 0 varies like *E* ^0.55^ to *E* and the corresponding differential flux *J*(*E*) varies like *E* ^3/4^ to *E* from 0.3 to 5 MeV. With significant electron loss (for *ϵ* ∼ 0.7), this steady‐state *F*(*E*) varies like *E* ^1/3^ to *E* ^3/4^ and the corresponding steady‐state *J*(*E*) varies like *E* ^1/2^ to *E* ^3/4^ from 0.3 to 5 MeV. Note that the hard energy spectra of these extreme steady‐state distributions still correspond to a decreasing electron PSD toward higher energy (*∂f*(*p*)/*∂p* < 0), allowing electron energy diffusion by chorus waves to continuously supply new electrons at higher energies (Walt, 1994), potentially maintaining the steady‐state shape of *F*(*E*) and *J*(*E*) in the presence of sufficient electron injections at low energy.

**Figure 1:**
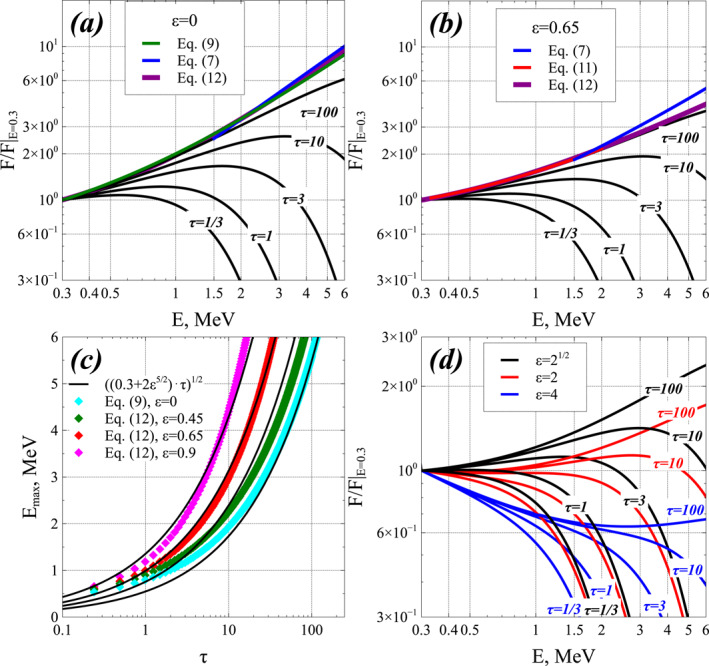
(a) Electron distributions *F*(*E*, *t*) obtained from numerical solution of the full Fokker‐Planck Equation 1 with fixed boundary conditions *F*(*E*_0_, *t*) = *F*(*E*_0_, *t* = 0) at *E*_0_ = 300 keV (corresponding to sustained low‐energy electron injections) and *F*(*E*_max_) = 0 at the upper energy boundary *E*_max_ = 100 MeV for 1/*τ*_*L*_ = 0 = *ϵ*, in black, and analytical steady‐state solutions given by Equations (7), (9) and 12 for *b* = 0, in blue, green, and purple, respectively, in their domain of validity. These conditions correspond to extreme geomagnetic activity with *Kp* > 7 and *AE* > 1,200 nT. The dimensionless time is $\tau =t\cdot \left(D_{EE}/E^{2}\right){|}_{1\;MeV}$. Simulation results are shown at *τ* = 1/3, 1, 3, 10, 100, corresponding here to *t* ≃ 0.04, 0.1, 0.4, 1, 10 days. Analytical steady‐state solutions are normalized to *F*(*E*) from simulations for *τ* = 100 at the lower energy limit of their domain of validity. (b) Same as (a) but for *ϵ* = 0.65 (corresponding to very strong disturbances with *Kp* > 5–6 and *AE* > 600 nT), with analytical steady‐state solutions given by Equations (7), (11), and 12 for *b* = 0 shown in blue, red, and purple, respectively. (c) Maximum energy *E*_max_ (cyan circles) where the numerical solution is within less than 30% of steady‐state solutions given by Equation 9 for *ϵ* = 0 and by Equation 12 for *ϵ* = 0.45, 0.65, 0.9, normalized at 0.3 MeV to the numerical solution at *τ* = 100. The best fit $E_{\max}≃\sqrt{\left(0.3+2\epsilon^{5/2}\right)\tau }$ is shown in black. (d) Electron distributions *F*(*E*, *t*) obtained from numerical solution of the full Fokker‐Planck Equation 1 with fixed boundary condition *F*(*E*_0_, *t*) = *F*(*E*_0_, 0) at *E*_0_ = 300 keV, for *ϵ* = 2^1/2^ (black), *ϵ* = 2 (red), and *ϵ* = 4 (blue), corresponding to faster electron loss than acceleration, most common during moderately active periods with *AE* < 400 nT or *Kp* < 4.

Figures 1a and 1b show comparisons between electron distributions *F*(*E*, *t*) (normalized to $F\left(E_{0}=0.3\text{MeV},t\right)$ obtained from numerical solution of the full one‐dimensional Fokker‐Planck diffusion Equation 1 and analytical steady‐state solutions given by Equations (7), (9), (11), and 12 for *b* = 0. In simulations, a fixed boundary condition *F*(*E*_0_, *t*) = *F*(*E*_0_, *t* = 0) at *E*_0_ = 0.3 MeV is adopted, corresponding to sustained low‐energy electron injections. In all simulations in this paper, we also use a realistic fixed condition *F*(*E*_max_) = 0 at the upper energy boundary *E*_max_ = 100 MeV, where electron fluxes are always negligible in the outer radiation belt. Although a constant *F*(*E*_0_, *t*) = *F*(*E*_0_, *t* = 0) with *F*(*E* > *E*_0_, *t* = 0) = 0 is not a solution to Equation 1 at *t* = 0, the *F*(*E*) gradient near *E*_0_ is assumed to very quickly relax initially, leading to a self‐consistent evolution given by Equation 1. Approximate analytical expressions for *τ*_*L*_ and *D*_*EE*_/*E* ^2^ given by Equations A5 and A6 in the work by Mourenas, Artemyev, Agapitov, Krasnoselskikh, and Li (2014), extensively validated by comparisons with numerical calculations (O. V. Agapitov et al., 2019; Artemyev, Mourenas, et al., 2013; Mourenas, Artemyev, Agapitov, & Krasnoselskikh, 2012), are employed in simulations. We adopt typical wave and plasma parameters during very strong disturbances with *Kp* > 5–6 and *AE* > 600 nT in the night/morning sector at low latitudes *λ* ≤ 10° and *L* = 5 (used to evaluate *D*_*EE*_): quasi‐parallel lower‐band chorus wave time‐ and MLT‐averaged root‐mean‐squared amplitude *B*_*w*_ = 120 pT, average normalized wave frequency *f*_*m*_/*f*_*ce*_ = 0.3, wave frequency spread Δ*f*/*f*_*m*_ ∼ 0.5, wave‐normal angle distribution width Δ*θ* ∼ 20°, and *f*_*pe*_/*f*_*ce*_ = 2 (O. V. Agapitov et al., 2018, 2019).

Comparisons between simulation results (in black) and analytical steady‐state solutions (in colors) in Figures 1a and 1b show a good agreement over their respective domains of validity. For negligible electron loss, *ϵ* = 0, the approximate analytical steady‐state solutions given by Equation 7, valid at *E* > 1.5 MeV, and given by Equation 12, valid for all *E*, are almost indistinguishable from the exact steady‐state solution given by Equation 9. These steady‐state solutions are very close to the asymptotic steady‐state distribution *F*(*E*, *t*) reached in simulations at a normalized time $\tau =t\cdot \left(D_{EE}/E^{2}\right){|}_{1MeV}=100$ up to ∼3 MeV. For significant electron loss, *ϵ* = 0.65, the analytical steady‐state solution given by Equation 12 is similarly very close to the asymptotic steady‐state distribution *F*(*E*) obtained in simulations at *τ* = 100 over the whole energy range 0.3–6 MeV.

The simulations in Figures 1a and 1b show that such steady‐state solutions should be considered as *time‐asymptotic solutions*, or limiting solutions, because the slower variation of *F*(*E*, *t*) as it approaches such stationary states (where *∂F*/*∂t* = 0) allows to reach them only after a sufficiently long period of sustained low‐energy electron injections and strong chorus‐driven acceleration. For the considered parameters, it requires $\Delta t∼\left(50-100\right)\times 1/D_{EE}$ (1 MeV), corresponding to ∼5–10 days of very strong geomagnetic activity with *Kp* > 5–6 and *AE* > 600 nT. Accordingly, the steady‐state electron distribution *F*(*E*) given by Equation 12 with *b* = 0 represents an attractor for the system dynamics (Lichtenberg & Lieberman, 1983), and likely corresponds to the hardest energy spectrum ever expected to be reached in the outer radiation belt—at least, in the absence of other physical processes.

These steady‐state solutions (Equations (9), (10), (11), (12)) for the distribution of radiation belt electrons are simply the result of a fine balance, in each energy range, between electron acceleration from lower energy that brings in new electrons, and electron acceleration to higher energy or loss to the atmosphere that removes electrons from this energy range. The corresponding slope *∂f*/*∂p* of the electron phase space density finely tunes the efficiency of electron diffusion toward higher energy (Schulz & Lanzerotti, 1974) to maintain this balance between incoming and outgoing electrons, at all energies comprised between some low energy *E*_0_ boundary fixed by electron injections and a maximum energy *E*_max_.

This maximum energy *E*_max_, where the steady‐state solution is nearly reached, increases over time in Figures 1a and 1b because electron acceleration requires more time to reach a higher energy (Horne et al., 2005). For a Dirac‐like instantaneous injection at *t* = 0 and no further injection, the upper energy limit *E*_*up*_ of significant acceleration corresponds to *∂F*(*t*)/*∂t* = 0 in Equation 2, giving *t*_max_(*E*_*up*_) ∼ *t*. For *E* ∈ [1, 6] MeV and *AE* > 600 nT, we usually have *ϵ* < 1 (O. V. Agapitov et al., 2019) and we can use the approximations *t*_max_(*E*_*up*_) ≃ *E* ^2^/(6 *D*_*EE*_ *τ*_*L*_) and $D_{EE}∼D_{EE}\left[1\right.$ MeV], giving $E_{up}∼\sqrt{6\;D_{EE}\left[1\;MeV\right]\;t}∼\sqrt{6\tau }$. But in the case of continuous injections and fixed *F*(*E*_0_), Figures 1a and 1b show that at any time *τ*, the electron distribution *F*(*E*, *t*) is still increasing at *E* = *E*_*up*_ on the right shoulder of the distribution. Equation 2 remains valid there, and it corresponds to a steeply decreasing shoulder to *F*(*E*) which broadens over time.

Nevertheless, we can use numerical results in Figures 1a and 1b to provide an estimate of the maximum energy *E*_max_ where the steady‐state solution is nearly reached, that is, where *F*(*E*, *τ*) is within less than 30% from the analytical steady‐state solutions (Equation 9 or Equation 12), normalized at 0.3 MeV to the numerical solution at *τ* = 100. Assuming the same scaling of *E*_max_ with $\sqrt{t}$ as for *E*_*up*_—which corresponds to a diffusive energy broadening of the distribution (e.g., see Balikhin et al., 2012; Mourenas, Artemyev, Agapitov, & Krasnoselskikh, 2014)—, it gives a best fit $E_{\max}≃\sqrt{\eta \;D_{EE}\left[1\;MeV\right]\;t}=\sqrt{\eta \;\tau }$ with *η* = 0.3 + 2*ϵ* ^5/2^, in good agreement with numerical results for all energies and 0 ≤ *ϵ* ≤ 1 in Figure 1c.

Finally, Figure 1d shows the temporal evolution of the electron distribution *F*(*E*, *t*) calculated numerically for *ϵ* = 2^1/2^, 2, and 4. Such high *ϵ* values correspond to a chorus wave‐driven electron precipitation that is faster than electron acceleration below 1 MeV. As expected, the negative first term in Equation 8 at low energy when $\epsilon >\sqrt{2}$ leads to a decreasing steady‐state *F*(*E*) (reached at *τ* = 100) up to ∼0.5–1 MeV and a much weaker increase at higher energy than for *ϵ* < 1. For $\epsilon >\sqrt{2}$, electron losses become sufficiently fast to prevent electron acceleration from increasing *F*(*E*) up to *F*(*E*_0_) below 1 MeV. This situation should be mainly encountered when *AE* < 400 nT or *Kp* < 4 (O. V. Agapitov et al., 2018, 2019). For 1 < *ϵ* < 2, a rough fit to the numerically obtained steady‐state *F*(*E*) in Figure 1d is given by Equation 12 with *ϵ* replaced by *ϵ* ^2/5^. Hua et al. (2022) have recently obtained a similar steady‐state solution as in Figures 1b and 1d, with a flux *J*(*E*) increasing with energy above ∼0.4 MeV, by numerically solving the full Fokker‐Planck equation in energy and pitch‐angle space, without any approximation, for a fixed set of realistic chorus wave and plasma parameters.

### 2.4 Dependence of Steady‐State Solutions on Wave and Plasma Parameters and Geomagnetic Activity

For *ϵ* = 0 (i.e., 1/*τ*_*L*_ = 0), the lowest steady‐state electron distribution solution is fully determined by Equation 9 with *b* = 0 and $a=F\left(E_{0}\right)/\left[\left(2E_{0}+1\right){\left(E_{0}\left(E_{0}+1\right)\right)}^{1/2}\left(\ln\left(E_{0}+1\right)-\ln\left(E_{0}\right)\right)\right]$, with *F*(*E*_0_) a fixed low‐energy boundary condition corresponding to injections. For 0 ≤ *ϵ* < 1 and *E*_0_ ≥ 0.3 MeV, the lowest steady‐state solution is fully determined by Equation 12 with *b* = 0 and $a=F\left(E_{0}\right)/{\left(2E_{0}+1\right)}^{\kappa }$ with $\kappa =3/2-{\left[3/16+10\epsilon^{2}/9\right]}^{1/2}$. Their domain of validity at a given time *t* is *E*_0_ ≤ *E* < *E*_max_, with $E_{\max}={\left[\left(0.25+2\epsilon^{5/2}\right)D_{EE}\left[1\;MeV\right]\;t\right]}^{1/2}$. Therefore, these steady‐state solutions only depend on *F*(*E*_0_), *D*_*EE*_[1 MeV], and *ϵ*.

Let us examine the dependence of the general steady‐state solution given by Equation 12 on wave and plasma parameters. Based on previous analytical estimates, validated against numerical simulations, the quasi‐linear chorus‐driven electron energization rate can be written as $D_{EE}\left[1\;MeV\right]≃50\;B_{w,acc}^{2}f_{ce}^{3/2}f_{m,acc}^{1/2}/f_{pe,acc}^{3}$ day^−1^ (Mourenas, Artemyev, Agapitov & Krasnoselskikh, 2012, 2014), with $B_{w,acc}^{2}$ (in pT^2^) the average chorus wave power at the low magnetic latitudes *λ* = 0°–10° of cyclotron resonance with accelerated, high equatorial pitch‐angle electrons (O.V. Agapitov et al., 2018, 2019; Aryan et al., 2020), *f*_*m*,*acc*_ and *f*_*pe*,*acc*_ the average wave frequency and plasma frequency over the local times of peak wave power at such low latitudes, and *f*_*ce*_ the equatorial gyrofrequency. The average wave normal angle distribution width is Δ*θ* ∼ 30° for quasi‐parallel chorus waves at low latitudes, although it may decrease to Δ*θ* ∼ 20° (O. V. Agapitov et al., 2015a, 2018).

Albert and Shprits (2009) have shown that the lifetime *τ*_*L*_ of electrons interacting with chorus waves can be written approximately as $\tau_{L}≃\sigma \int_{\alpha_{LC}}^{90{}^{\circ}}d\alpha /\left(2\;D_{\alpha \alpha }\tan\;\alpha \right)$, with *D*_*αα*_ the quasi‐linear electron pitch‐angle diffusion rate, *α*_*LC*_ the equatorial loss‐cone angle, and *σ* ≃ 0.5–1 a numerical coefficient allowing to recover precisely the lifetime value obtained from full numerical simulations (e.g., see Albert & Shprits, 2009; Artemyev, Mourenas, et al., 2013). This formulation clearly indicates that the main contribution to *τ*_*L*_ comes from the *α*‐region where (*D*_*αα*_ tan *α*) is minimum (Albert & Shprits, 2009). Based on previous analytical estimates validated by numerical simulations, we have also obtained $D_{\alpha \alpha }\left[1\;MeV\right]≃1.9\;B_{w,loss}^{2}f_{ce}^{4/3}/\left(f_{m,loss}^{7/9}f_{pe,loss}^{14/9}\cos^{2}\alpha \right)$ day^−1^ for *L* ∼ 5 and *α* < 60°, where (*D*_*αα*_ tan *α*) is minimum, corresponding to *σ* ≃ 0.7 (O. V. Agapitov et al., 2019; Artemyev, Mourenas, et al., 2013; Mourenas, Artemyev, Ripoll, et al., 2012). Here, $B_{w,loss}^{2}$, *f*_*m*,*loss*_, and *f*_*pe*,*loss*_ denote, respectively, the chorus wave power, wave frequency at peak power, and plasma frequency at peak wave power (e.g., see O. V. Agapitov et al., 2018, 2019), averaged over MLT at magnetic latitudes *λ* ∼ 15°–35° of cyclotron resonance with ∼0.3–3 MeV electrons near the loss‐cone, which are precipitated into the atmosphere by chorus waves (O. V. Agapitov et al., 2018; Artemyev, Mourenas, et al., 2013). This finally gives *τ*_*L*_ ≃ 0.8/*D*_*αα*_(*α*_*LC*_) for ∼1 MeV electrons at *L* ∼ 5. Substituting in *ϵ* = 2^5/4^(*E* ^2^/(*D*_*EE*_ *τ*_*L*_))|_1MeV_ the above approximate analytical expressions of *D*_*EE*_[1 MeV] and *τ*_*L*_[1 MeV], we get

(13) $$\epsilon ≃\frac{1}{9}{\left(\frac{B_{w,loss}}{B_{w,acc}}\right)}^{2}{\left(\frac{f_{pe,acc}}{f_{pe,loss}}\right)}^{3/2}{\left(\frac{f_{pe,acc}}{f_{ce}}\right)}^{3/2}{\left(\frac{f_{ce}}{f_{m,loss}}\right)}^{7/9}{\left(\frac{f_{ce}}{f_{m,acc}}\right)}^{1/2}.$$

Alternatively, when full measurements of wave and plasma parameters are not available, one can use an empirical estimate of *ϵ* as a function of geomagnetic activity *AE*. Statistics of bounce‐ and MLT‐averaged diffusion rates *D*_*EE*_ and *D*_*αα*_(*α*_*LC*_) of 1‐MeV electrons by lower‐band chorus waves, calculated based on simultaneous measurements of chorus waves and plasma density by the Van Allen Probes in 2012–2017, have indeed shown that *ϵ* varies with *AE* ∈ [50, 1,500] nT at *L* ∼ 4–6 approximately as (O. V. Agapitov et al., 2019):

(14) $$\epsilon \;\approx \;\frac{2700}{\;{\left(AE\right)}^{5/4}}.$$

The above scaling laws show that at a given electron energy *E* ∼ *E*_max_, the steady‐state spectrum is more rapidly reached for a shorter electron lifetime *τ*_*L*_[1 MeV], in agreement with numerical results in Figure 1c. This corresponds to a higher chorus wave power and a lower plasma density at middle/high latitudes in the morning/day sector (O. V. Agapitov et al., 2018). However, a shorter electron lifetime *τ*_*L*_[1 MeV] also corresponds to a higher *ϵ* and a softer electron energy spectrum (i.e., a less steep increase of *F*(*E*) with *E*) than for a longer lifetime and a smaller *ϵ* (see Equation 12 and compare Figures 1a and 1b). In other words, faster precipitation losses restrain electron acceleration and decrease the maximum steady‐state electron flux that can be attained, although they simultaneously reduce the time needed to reach it. In reality, the efficiency of electron acceleration to higher *E* is mainly controlled by *D*_*EE*_[1 MeV]. For a fixed electron lifetime *τ*_*L*_[1 MeV], the steady‐state electron energy spectrum is harder for a smaller *ϵ*, corresponding to a higher *D*_*EE*_[1 MeV]. For a fixed *ϵ* = 2^5/4^/(*τ*_*L*_ *D*_*EE*_)|_1MeV_, the steady‐state electron energy spectrum is also more rapidly reached for a higher *D*_*EE*_[1 MeV]. A higher *D*_*EE*_[1 MeV] corresponds to higher chorus wave power, wave frequency, and lower plasma density, at low latitudes in the midnight/morning sector.

Since the hardest steady‐state electron energy spectra correspond to a smaller *ϵ* in Equation 12, Equation 13 further shows that they should be reached when the low‐latitude to high‐latitude chorus wave power ratio ${\left(B_{w,acc}/B_{w,loss}\right)}^{2}$ is higher, the midnight/morning to morning/day plasma density ratio ${\left(f_{pe,acc}/f_{pe,loss}\right)}^{2}$ is smaller, and both the low‐latitude and high‐latitude average normalized wave frequencies *f*_*m*_/*f*_*ce*_ are larger. This occurs during particularly disturbed conditions, with high *AE* > 500 nT and *Kp* > 4, which usually lead to plasmasphere erosion and to a strong reduction of plasma density in the night sector (O. V. Agapitov et al., 2019), where are simultaneously injected more abundant populations of 3–30 keV electrons most efficient for generating lower‐band chorus waves (Li et al., 2010). The lower *f*_*pe*,*acc*_/*f*_*ce*_ ratio in the night sector strongly increases electron diffusive acceleration by chorus waves (O. V. Agapitov et al., 2018, 2019; Summers et al., 1998) and may allow the electron distribution to reach its steady‐state.

However, most geomagnetically active periods of time‐averaged *AE* higher than 400 nT, corresponding to sustained injections and strong chorus wave growth, last less than 3 days (Mourenas et al., 2019; Mourenas, Agapitov, et al., 2022). Consequently, the extreme asymptotic steady‐state regime given by Equation 12 with *b* = 0 and *ϵ* < 1 should not be attained frequently. But it could be reached, at least at *E* < 3 MeV, during the periods of highest time‐integrated *ap* and *AE* and continuously high *Kp* > 3, as during the long 9–17 November 2003 event with an average *AE* of 580 nT, or during the 5–14 February and 2–14 April 1994 events with average *AE* of ∼500 nT (Mourenas et al., 2019). Such extreme events often contain high‐intensity long‐duration continuous auroral activity (HILDCAA) episodes produced by high‐speed solar wind streams (Tsurutani et al., 2006), as in November 2003. During such extended periods of sustained low energy electron injections, the steady‐state electron distribution *F*(*E*) given by Equation 12 with *b* = 0 should represent an attractor for the outer radiation belt dynamics, because *F*(*E*, *t*) varies much more slowly in its vicinity and also because it is the first state of this kind that can be reached from an initial condition with low electron flux at high energy. Such a steady‐state electron distribution should correspond to the hardest energy spectrum that can be encountered over prolonged periods in the outer radiation belt. It could be reached only during the most extreme and sustained geomagnetic events, with a high time‐integrated geomagnetic activity (Mourenas et al., 2019; Mourenas, Agapitov, et al., 2022).

### 2.5 Numerical Investigation of Propitious Conditions for Reaching a Steady State: Importance of Electron Injections and Plasma Density

The steady‐state solutions given by Equations (9), (10), (11), (12) to the Fokker‐Planck Equation 1 can be reached during prolonged disturbed periods. But this necessarily requires sufficiently strong and sustained low energy electron injections from the plasma sheet to keep *F*(*E*_0_) nearly constant for a sufficiently long time (Bakhareva, 2005; Hua et al., 2022; Summers et al., 2002), providing both an anchor point for the steady‐state electron distribution and the inflow of energetic particles needed to maintain the electron flux level practically unchanged over a wide energy range despite the continuous acceleration and loss. Is it realistic?

Based on Equation 2, the presence of a constant electron distribution *F*(*E*_0_, *t*) = *F*(*E*_0_, *t* = 0) at low energy *E*_0_ ∼ 100–300 keV and *L* = 4–6.5 outside the plasmasphere requires an injection rate ${\left(\partial F\left(E_{0}\right)/\partial t\right)}_{\mathrm{inj}}>F\left(E_{0},t=0\right)/\tau_{L}$ as *t* increases above 3*t*_max_ ∼ *τ*_*L*_/2, with *τ*_*L*_ ∼ 3–10 hr at 100–300 keV during active periods with *Kp* > 3.5 (O. V. Agapitov et al., 2018). Such timescales correspond to typical timescales of substorm‐related electron injections in the outer radiation belt (Arnoldy & Chan, 1969; Gabrielse et al., 2014; Meredith et al., 2000, 2002). Although individual injections occur over ≈2–10 min, series of injections often occur over ∼1–5 hr during substorms (Birn et al., 1997; Gabrielse et al., 2014), and they can indeed persist several days during periods of prolonged substorm activity (high *AE*) in association with enhanced chorus wave amplitudes (Meredith et al., 2002, 2003; Tang et al., 2017), maintaining a roughly constant electron flux level at *E*_0_ ∼ 100–300 keV over ∼3–5 days at *L* ∼ 4.5–6.5 in the outer belt (Hua et al., 2022; Murphy et al., 2018), with the help of the Kennel‐Petschek flux limitation mechanism very efficient at such low energies (Kennel & Petschek, 1966; Olifer et al., 2021; Summers & Shi, 2014).

**Figure 2:**
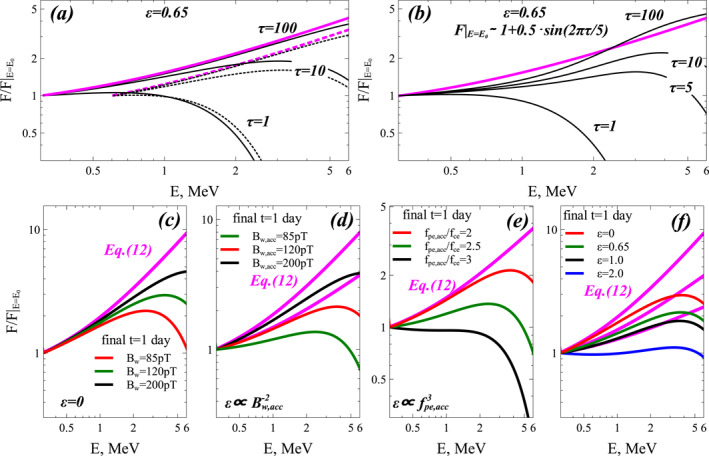
(a) Variation of normalized electron distribution *F*(*E*, *t*)/*F*(*E*_0_) obtained from numerically solving the Fokker‐Planck diffusion Equation 1 with fixed boundary conditions, *F*(*E*_0_, *t*) = *F*(*E*_0_, *t* = 0) for *E*_0_ = 300 keV (solid black) and *E*_0_ = 600 keV (dashed black) and *F*(*E*_max_) = 0 at the upper energy boundary *E*_max_ = 100 MeV, and *ϵ* = 0.65. Analytical steady‐state solutions given by Equation 12 with *b* = 0 are shown when they are available, that is, when 0 ≤ *ϵ* ≤ 1 (purple). The dimensionless time is $\tau =t\cdot \left(D_{EE}/E^{2}\right){|}_{1MeV}$ and wave and plasma parameters are the same as in Figure 1. (b) Same as (a) but for *E*_0_ = 300 keV and a varying boundary condition *F*(*E*_0_, *t*) = *F*(*E*_0_)[1 + 0.5 sin(2*πτ*/5)] in the simulation (black), compared to analytical steady‐state solution given by Equation 12 for *F*(*E*_0_, *t*) = *F*(*E*_0_) (purple). (c) Variation of *F*(*E*, *t*)/*F*(*E*_0_) for *E*_0_ = 300 keV at a fixed final time *t* = 1 day in all simulations, as a function of MLT‐averaged chorus wave power $B_{w}^{2}$ at all latitudes, for *ϵ* = 0. (d) Same as (c) but for a varying chorus wave power $B_{w,acc}^{2}$ only at low latitudes (corresponding to varying $\epsilon ∼1/D_{EE}∼1/B_{w,acc}^{2}$), taking *ϵ* = 0.72 for *B*_*w*,*acc*_ = 120 pT. (e) Same as (c) but as a function of *f*_*pe*,*acc*_/*f*_*ce*_ at low latitudes on the nightside (corresponding to varying $\epsilon ∼1/D_{EE}∼f_{pe,acc}^{3}$), taking *ϵ* = 0.72 for *f*_*pe*,*acc*_/*f*_*ce*_ = 2. (f) Same as (c), but as a function of *ϵ*.

Figure 2 presents a numerical investigation, using Equation 1, of the relative importance of the different wave and plasma parameters for reaching the steady‐state electron distribution given by Equation 12 within a fixed laps of time. The nominal simulation parameters are the same as in Figure 1. They correspond to very strong disturbances at *L* = 5, with *Kp* > 5–6 and *AE* > 600 nT: root‐mean‐squared lower‐band chorus wave amplitude *B*_*w*,*acc*_ = 120 pT and *f*_*pe*,*acc*_/*f*_*ce*_ = 2 at low latitudes on the night/morning side, *ϵ* = 0.65 (O. V. Agapitov et al., 2018, 2019), and a constant *F*(*E*_0_, *t*) at the low energy boundary *E*_0_ = 300 keV. We examine the impact of these different parameters in the six panels of Figure 2.

Figure 2a shows the variation of the normalized *F*(*E*, *t*)/*F*(*E*_0_) as the maximum energy *E*_0_ of electron injections is increased from 300 to 600 keV, demonstrating that the energy spectrum *F*(*E*, *t*) evolution is very similar in both cases. Figure 2b displays the evolution of $F\left(E,t\right)/{\langle F\left(E_{0},t\right)\rangle }_{t}$ for a strongly oscillating low‐energy boundary condition *F*(*E*_0_, *t*) = *F*(*E*_0_)[1 + 0.5 sin(2*πτ*/5)] (black curves). A comparison with the analytical steady‐state solution given by Equation 12 with *b* = 0 for a constant *F*(*E*_0_, *t*) = *F*(*E*_0_) (purple curve), and with numerical results for constant *F*(*E*_0_, *t*) in Figure 1b, shows that the evolution of $F\left(E,t\right)/{\langle F\left(E_{0},t\right)\rangle }_{t}$ is robust, almost insensitive over the long term to ordinary fluctuations of *F*(*E*_0_) due to a fluctuating intensity of electron injections.

In Figure 2c, the average chorus wave power $B_{w}^{2}$ is varied equally at all latitudes, corresponding to a constant *ϵ* but a varying energy diffusion rate $D_{EE}∼B_{w}^{2}$ of electrons by chorus waves. In this case, the normalized *F*(*E*, *t*) increases faster and comes more rapidly close to the steady‐state solution for a higher $B_{w}^{2}$, as expected. In Figure 2d, only the average chorus wave power $B_{w,acc}^{2}$ at low latitudes is varied, whereas the high‐latitude chorus power $B_{w,loss}^{2}$ is kept constant, corresponding to varying $\epsilon ∼1/D_{EE}∼1/B_{w,acc}^{2}$. In this case, a higher $B_{w,acc}^{2}$ similarly leads to a faster increase of the normalized *F*(*E*, *t*), allowing to reach the steady‐state solution more rapidly. However, this steady‐state *F*(*E*) level simultaneously becomes higher at higher $B_{w,acc}^{2}$, than when the low‐latitude to high‐latitude chorus power ratio $B_{w,acc}^{2}/B_{w,loss}^{2}$ is kept fixed, because—as indicated by—the increase of $B_{w,acc}^{2}$ corresponds to a reduced *ϵ* in Equation 12.

Figure 2e shows the effect of varying the plasma frequency to gyrofrequency ratio *f*_*pe*,*acc*_/*f*_*ce*_ at low latitudes on the nightside, equivalent to varying $\epsilon ∼1/D_{EE}∼f_{pe,acc}^{3}$. Equation 13 shows that *ϵ* varies like $\epsilon ∼{\left(B_{w,loss}/B_{w,acc}\right)}^{2}f_{pe,acc}^{3}$, where subscripts “acc” and “loss” denote the regions of cyclotron resonant acceleration and precipitation of electrons, located respectively at low latitude in the night sector and at middle/high latitude on the dayside (O. V. Agapitov et al., 2018; Mourenas, Artemyev, Agapitov, & Krasnoselskikh, 2014; Mourenas, Artemyev, Agapitov, Krasnoselskikh, & Li, 2014). Thus, similar variations of $B_{w,acc}^{2}$ and $1/f_{pe,acc}^{3}$ are expected to have a similar effect on *F*(*E*, *t*)/*F*(*E*_0_). This is confirmed by Figure 2e where a lower *f*_*pe*,*acc*_/*f*_*ce*_, like a higher $B_{w,acc}^{2}$ in Figure 2d, leads to a faster increase of *F*(*E*, *t*) and a higher steady‐state *F*(*E*) level, more rapidly reached. These two parameters, $B_{w,acc}^{2}$ and *f*_*pe*,*acc*_/*f*_*ce*_, are the main wave and plasma parameters controlling both the maximum level of the steady‐state electron distribution *F*(*E*) and flux *J*(*E*) and the likelihood of reaching this steady‐state *F*(*E*) at high energy during a fixed and realistic laps of time.

### 2.6 Uncertainties of Analytical Steady‐State Solutions

The electron flux corresponding to the analytical steady‐state solutions in Equations 9 and 12 only depends on *F*(*E*_0_) and *ϵ*, while the time scale for reaching it depends on *D*_*EE*_(1 MeV). The analytical estimates of *D*_*EE*_ and *τ*_*L*_ used here usually remain within less than a factor of ∼1.5–2 from exact numerical values above 0.3 MeV for *f*_*pe*_/*f*_*ce*_ ≥ 2 (O. V. Agapitov et al., 2019; Artemyev, Mourenas, et al., 2013; Aryan et al., 2020; Mourenas, Artemyev, Agapitov, & Krasnoselskikh, 2012), resulting in a factor of ≈2 uncertainty for the analytical estimate in Equation 13 of *ϵ*. The estimate in Equation 14 giving *ϵ* as a function of *AE* based on chorus wave and plasma density statistics, has a similar uncertainty (O. V. Agapitov et al., 2019). Equation 12 further shows that the steady‐state *F*(*E*) varies less than linearly with *ϵ* below ∼3–4 MeV, giving a steady‐state *F*(*E*)/*F*(*E*_0_) uncertainty smaller than *ϵ* uncertainty. Since the evolution of *F*(*E*, *t*) is very slow near the steady state, the exact *F*(*E*, *t*)/*F*(*E*_0_) should remain close to the analytical steady‐state estimate at *E* < *E*_max_.

In a given *AE* or *Kp* range, the chorus wave power latitudinal distribution varies significantly with time and MLT, due to the variability of wave growth and damping provided by injections of anisotropic 1–50 keV electron populations (L. Chen et al., 2013; Li et al., 2010), which could affect *ϵ* through a change of the high‐latitude to low‐latitude wave power ratio in Equation 13. *ϵ* may also vary with plasma density, which determines the latitude of cyclotron resonance for precipitated electrons. But during events lasting more than ∼2–3 days, the time‐averaged and MLT‐averaged chorus distribution and plasma density, which mainly determine the cumulative electron energization and loss, should remain close to their average distributions inferred from multi‐year satellite statistics.

Last but not least, the additional presence of intense EMIC or ULF waves may modify the steady‐state solution *F*(*E*) (Li et al., 2007; Mourenas et al., 2016; Ozeke et al., 2020; Ross et al., 2021; Summers & Thorne, 2003). The potential effects of EMIC and ULF waves are examined in the next sections.

## 3 Analytical Steady‐State Electron Distribution in the Presence of Chorus and EMIC Waves

In Section 2, we assumed the presence of intense chorus waves above the plasmapause, with only weak contemporaneous EMIC waves in a high‐density plasmaspheric boundary or plume region in the dusk sector with time‐averaged and MLT‐averaged EMIC magnetic power $B_{w}^{2}$ near the equator smaller than ∼1/5−1/10 of the time‐averaged and MLT‐averaged chorus wave power at the latitudes *λ* > 25° of cyclotron resonance between chorus waves and >1.5 MeV electrons near the loss‐cone. In such a situation, EMIC waves should not significantly modify electron loss rates driven by chorus waves (Mourenas et al., 2016). However, Van Allen Probes statistics of chorus and EMIC waves during disturbed periods with *Kp* > 3–4 or *AE* > 400 nT (and solar wind dynamic pressure *Pdyn* ≥ 2 nPa) rather show similar average hydrogen band EMIC and chorus wave powers in these two respective latitudinal ranges, or even a higher EMIC wave power (O. V. Agapitov et al., 2018, 2019; Ross et al., 2021; X.‐J. Zhang et al., 2016). Substorm‐related particle injections from the plasma sheet indeed provide both low‐energy electron and ion populations with high temperature anisotropies, which may respectively generate chorus and EMIC waves (Birn et al., 2014; Kennel & Petschek, 1966). But while chorus waves are preferentially excited in low density regions in the night/dawn sector (O. V. Agapitov et al., 2018; Horne et al., 2005; Meredith et al., 2002), EMIC waves are preferentially excited in the plasmasphere boundary/plume region on the dusk side (H. Chen et al., 2020; L. Chen et al., 2014; Cornwall et al., 1970; Kozyra et al., 1997; Ross et al., 2021), or around noon during solar wind dynamic pressure enhancements (H. Chen et al., 2020; Olson & Lee, 1983; Ross et al., 2021).

Although individual EMIC wave bursts usually last less than several hours and are confined in narrow MLT domains (O. V. Agapitov et al., 2017; Ross et al., 2021), their long‐term effects on electron lifetimes, over periods longer than a few days, can be modeled by statistical time‐ and MLT‐averaged quasi‐linear pitch‐angle diffusion rates (Mourenas et al., 2021; Summers & Thorne, 2003), as in the case of hiss waves or VLF waves from transmitters (O. Agapitov et al., 2020; Ross et al., 2019). Very intense EMIC wave‐packets may lead to non‐linear effects (Grach et al., 2022), but the presence of mainly short EMIC wave‐packets separated by random wave frequency and phase jumps (e.g., see various examples of such short packets in Usanova et al. [2010] and X. An et al. [2022]) is expected to allow an approximate quasi‐linear diffusive treatment, as in the case of chorus wave‐packets (Artemyev et al., 2021, 2022; X. J. Zhang, Agapitov, et al., 2020).

In the presence of typical intense hydrogen band EMIC waves at frequencies up to *f* ∼ 0.45 *f*_*cp*_ (with *f*_*cp*_ the proton gyrofrequency) in a duskside plasmaspheric plume (X.‐J. Zhang et al., 2016) where *f*_*pe*_/*f*_*ce*_ ∼ 15–20 (Sheeley et al., 2001) at the same *L* ∼ 5–6 as dawnside chorus waves, electron lifetimes should be strongly reduced above a minimum energy E* ≈ 1.5 MeV of cyclotron resonance with EMIC waves, compared with lifetimes in the presence of chorus waves alone, as confirmed by satellite observations (Drozdov et al., 2020; Mourenas et al., 2016, 2021; X.‐J. Zhang et al., 2017). Although EMIC waves can rarely significantly scatter high equatorial pitch‐angle electrons at energies *E* < 5 MeV (Kersten et al., 2014; Ross et al., 2021), the contemporaneous presence of intense chorus waves indeed allows to fill their pitch‐angle diffusion trough at high pitch‐angles and to rapidly scatter high pitch‐angle electrons down to the loss‐cone (Mourenas et al., 2016). For a sufficiently high ratio ≳1 of hydrogen band EMIC wave power to chorus wave power (averaged at their respective latitudes of cyclotron resonance with multi‐MeV electrons near the loss‐cone) as in spacecraft statistics when *Kp* > 3–4 or *AE* > 400 nT (O. V. Agapitov et al., 2018, 2019; Ross et al., 2021; X.‐J. Zhang et al., 2016), the resulting lifetimes depend weakly on the EMIC wave power in the duskside plume for E* < *E* < 5 MeV and mainly depend on the chorus‐driven *D*_*αα*_(*α*_*LC*_) (Mourenas et al., 2016). In the same energy range, the resulting electron lifetimes are also nearly independent of *E*, until they increase again like ∼*E* ^2^ at higher energy (Mourenas et al., 2016, 2021). Since E* varies with EMIC wave frequency and local *f*_*pe*_/*f*_*ce*_ (Summers & Thorne, 2003), electron scattering by various EMIC waves with different frequencies over the course of several days, in both hydrogen and helium bands and in regions of different plasma densities (Ross et al., 2021; X.‐J. Zhang et al., 2016), should further weaken the dependence of the resulting lifetimes on electron energy (Mourenas et al., 2016; Ross et al., 2021).

Accordingly, during disturbed periods with *Kp* > 3–4 or *AE* > 400 nT, characterized by a sustained presence of intense EMIC waves as in Van Allen Probes statistics at *L* ∼ 4–6 (Ross et al., 2021; X.‐J. Zhang et al., 2016), the effective electron lifetime $\tau_{L}^{\mathrm{eff}}$ can be taken approximately as $\tau_{L}^{\mathrm{eff}}∼\tau_{L}\left(1\;MeV\right)/\kappa$ from *E* ≃ E* ≃ 1.5 MeV up to ∼5 MeV, with *τ*_*L*_ the lifetime due to chorus waves alone and *κ* ∼ 7–9 based on theory and observations (Mourenas et al., 2016, 2021; X.‐J. Zhang et al., 2017). The corresponding steady‐state solution to the Fokker‐Planck diffusion Equation 1 at *E* > 1.5 MeV is given by Equation 8, where *ϵ* is replaced by (2^1/4^ *κ*/3) (*E* + 1/2) (*E*(*E* + 1))^3/4^ *ϵ*. Here, *ϵ* is given by the empirical Equation 14 as a function of *AE*. Approximating the first five multiplication factors to *F* in Equation 8 by 32*E* ^2^(*E* + 1)^2^ and using the excellent approximation *E*(*E* + 1) ≃ (*E* + 1/2)^2^ at *E* > 1.5 MeV in all its terms, yields a differential equation with the following exact solution:

(15) $$F\left(E\right)\;≃\;a\cdot E\cdot \exp\left(-E\;\sqrt{B\;\epsilon \;}\;\;\right)$$

with *B* = 2^1/4^ *κ*/3 ≃ 3 − 4 and *a* a normalization constant, valid at *E* > 1.5 MeV. This type of stationary solution was first obtained by Bakhareva (2003, 2005) for constant *τ*_*L*_ (as here) and *D*_*EE*_, and the above approximation *E*(*E* + 1) ≃ (*E* + 1/2)^2^ is indeed equivalent to taking *D*_*EE*_ constant for quasi‐parallel chorus waves (Mourenas, Artemyev, Agapitov & Krasnoselskikh, 2012, 2014). At sufficiently low energy *E* < 0.5 MeV, the steady‐state solution should still be given approximately by Equation 12 when *ϵ* ≤ 1. However, the presence of this fast electron loss at *E* > E* ∼ 1.5 MeV represents an additional drainpipe for the full distribution *F*(*E*), necessarily leading to a decrease of its normalized steady‐state level at all energies, although this decrease should be more important at higher energy. As the steady‐state solution in Equation 15 has the interesting property of increasing from low energy up to $E=1/\sqrt{B\epsilon }<1.5$ MeV similarly to the solution in Equation 12 for *ϵ* ≤ 1, this suggests that an approximate steady‐state solution valid over the full energy range *E* ≥ 0.3 MeV, may be written as:

(16) $$F\left(E\right)\;≃\;F\left(E_{0}\right)\cdot {\left(\frac{E}{E_{0}}\right)}^{\alpha }\cdot \exp\left(\left(E_{0}-E\right)\;\alpha \;\sqrt{B\;\epsilon \;}\;\right),$$

with *B* ≃ 3–4 and $\alpha ≃\max\left(1/2,\tanh\left(E^{2}\right)\right)$. At *E* > 1.5 MeV, this expression for *α* gives *α* = 1, allowing to recover the solution in Equation 15 valid for *E* > 1.5 MeV. At lower energy *E* < 1.5 MeV, *α* decreases and reaches 1/2 below 0.75 MeV. This yields a higher ratio *F*(*E*)/*F*(*E*_0_) < 1 between 1.5 MeV and ∼1 MeV than in Equation 15, as expected in the presence of much slower electron loss at *E* < 1.5 MeV. Below 0.75 MeV, this also gives a *F*(*E*)/*F*(*E*_0_) > 1 ratio halfway between the solution in Equation 15, which is similar there to the solution in Equation 12 without EMIC waves, and the level *F*(*E*)/*F*(*E*_0_) = 1 at *E* = *E*_0_, as expected since the fast losses above 1.5 MeV should reduce the chorus‐driven increase of *F*(*E*) at all energies, although much less at lower energy. For *ϵ* ≫ 2^5/4^/*κ*, the steady‐state shape of *F*(*E*) is reached at $t>t_{\max}\left(E\right)\approx E/\left[\sqrt{2B\epsilon }D_{EE}\left(1\;MeV\right)\right]$ above 1.5 MeV (Artemyev, Agapitov, et al., 2013). At very high energy *E* > 5 MeV, the lifetime is expected to increase again with energy as $\tau_{L}^{\mathrm{eff}}\left(E\right)∼\tau_{L}\left(E\right)/20$ (Mourenas et al., 2016, 2021; X.‐J. Zhang et al., 2017), leading to a steady‐state *F*(*E*) given by Equation 7 with *ϵ* replaced by 20*ϵ* and *b* = 0.

**Figure 3:**
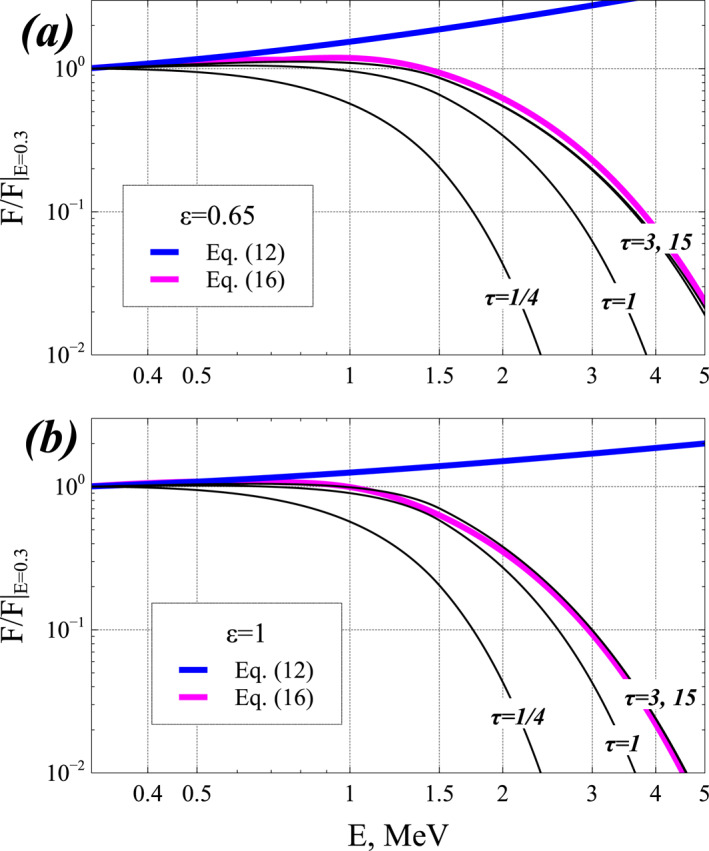
(a) Variation of normalized electron distribution *F*(*E*, *t*)/*F*(*E*_0_) obtained from numerically solving the Fokker‐Planck diffusion Equation 1 with fixed boundary conditions, *F*(*E*_0_, *t*) = *F*(*E*_0_, *t* = 0) at *E*_0_ = 300 keV and *F*(*E*_max_) = 0 at the upper energy boundary *E*_max_ = 100 MeV, and *ϵ* = 0.65, with *ϵ* multiplied by (2^1/4^7/3) (*E* + 1/2) (*E*(*E* + 1))^3/4^ at *E* > 1.5 MeV to take into account a faster electron loss caused by combined scattering by chorus and EMIC waves (black). The corresponding analytical steady‐state solutions given by Equation 12 with *b* = 0 (blue), in the presence of chorus waves but without EMIC waves, and by Equation 16 with *B* = 3 (purple), in the presence of chorus and EMIC waves, are shown. Typical wave and plasma parameters at *L* ∼ 5 when *AE* ∼ 500–600 nT or *Kp* ∼ 4–5 are used: *B*_*w*,*acc*_ ≃ 55 pT, *f*_*m*_/*f*_*ce*_ = 0.25, Δ*θ* = 30°, and *f*_*pe*,*acc*_/*f*_*ce*_ = 4, corresponding to *D*_*EE*_/*E* ^2^ ∼ 0.15 day^−1^ at 1 MeV (O.V. Agapitov et al., 2018, 2019) and $\tau_{L}^{\mathrm{eff}}≃3.5$ days above 1.5 MeV in the presence of intense chorus and EMIC waves. The dimensionless time is $\tau =t\cdot \left(D_{EE}/E^{2}\right){|}_{1\;MeV}$. Simulation results are shown at *τ* = 1/4, 1, 3, 15, corresponding to *t* ∼ 2, 6, 20, 100 days. (b) Same as (a) but for *ϵ* = 1, corresponding to $\tau_{L}^{\mathrm{eff}}≃2.2$ days above 1.5 MeV in the presence of chorus and EMIC waves.

Figure 3 shows comparisons between numerical simulation results, in black, and the approximate analytical steady‐state solution given by Equation 16, in purple, at *L* = 5 in the presence of intense EMIC and chorus waves in different MLT sectors. In striking contrast with simulation results without EMIC waves in Figure 1b and with the steady‐state solution in Equation 12 without EMIC waves (in blue), the much shorter lifetimes of >1.5 MeV electrons in the presence of combined scattering by EMIC waves at low equatorial pitch‐angles and by chorus waves at high pitch‐angles (Mourenas et al., 2016; X.‐J. Zhang et al., 2017) leads in Figure 3a to a steady‐state *F*(*E*) with a fast‐dropping shoulder above 1.5 MeV (black and purple curves). The steady‐state shape (in purple) is more rapidly reached than without EMIC waves, because accelerating a much smaller number of electrons to multi‐MeVs requires less time. The approximate analytical steady‐state solution given by Equation 16 is in very good agreement with numerical simulations for both *ϵ* = 0.65 and *ϵ* = 1 in Figures 3a and 3b, despite the faster decrease of *F*(*E*) above 2 MeV for higher *ϵ*.

The results in Figures 1b and 3a further suggest a novel method to assess the presence of EMIC waves, through the examination of electron flux energy spectra *J*(*E*) ≃ *F*(*E*) after *τ* > 1 during sufficiently long‐lasting events with *ϵ* ≤ 1—that is, after at least ∼6 days of realistically strong chorus wave‐driven electron energization with *D*_*EE*_/*E* ^2^ ∼ 0.15 day^−1^ at 1 MeV for an average *AE* ∼ 500–600 nT or an average *Kp* ∼ 4–5 (O. V. Agapitov et al., 2018, 2019; Thorne et al., 2013). Without EMIC waves, such long and intense events should indeed lead to a plateau of *F*(*E*) extending up to ∼3 MeV, whereas in the presence of EMIC waves, this plateau should end at ∼1.5 MeV. We shall come back to this point in Section 5. The corresponding effective lifetimes of >1.5 MeV electrons in the presence of intense chorus and EMIC waves in different MLT sectors are $\tau_{L}^{\mathrm{eff}}∼1.6-2.2$ days for *ϵ* = 1 and *B* = 3–4. Such effective lifetimes are consistent with the minimum lifetimes of 1.5–5 MeV electrons measured by ELFIN and LANL spacecraft at *L* ∼ 5.0–6.7 outside the plasmasphere (Boynton et al., 2014; Mourenas et al., 2021), and by the Van Allen Probes at *L* = 5.5–6.0 probably outside the plasmasphere (Claudepierre et al., 2020).

## 4 Influence of Electron Radial Diffusion by ULF Waves

Based on Time History of Events and Macroscale Interactions during Substorms (THEMIS) spacecraft statistics, the differential flux spectrum *J*(*E*) of energetic electrons, transported earthward within Dipolarizing Flux Bundle (DFB) channels and injected at *L* ∼ 7–9 just outside the outer radiation belt, has a typical shape *J*(*E*) ∼ 1/*E* ^2.5^ over 40–400 keV (Runov et al., 2015), corresponding to *f*(*p*) ∼ 1/*p* ^6.5^. Such electrons can be further diffused radially inward by ULF waves, potentially modifying the electron energy spectrum at relativistic energies. The radial diffusion rate *D*_*LL*_ due to ULF waves is weakly dependent on electron energy *E* and pitch angle *α* in the case of electrostatic ULF perturbations, usually dominant in *D*_*LL*_ (Ozeke et al., 2014). In this case, conservation of the first adiabatic invariant (Schulz & Lanzerotti, 1974) implies that the initial normalized energy spectrum *f*(*p*)/*f*(*p*_min_) of high *α* ≈ 90° electrons present at *L* = 7 − 9 should be conserved during their inward diffusive transport and acceleration by ULF waves when electron loss and chorus wave‐driven energization are negligible. This yields a power‐law energy spectrum *F*(*E*) ∼ (*E* + 1/2)/(*E*(*E* + 1))^2.75^ and a differential flux *J*(*E*) ∼ 1/(*E*(*E* + 1))^2.25^ at *L* = 4 − 6 for *E* > 0.25–0.35 MeV, close to the variation of the average *J*(*E*) over 0.2–3.0 MeV in the AE8 empirical model at geosynchronous orbit (Vette, 1991). This energy spectrum is much more rapidly decreasing toward high energy over 0.3–3 MeV than the steady‐state energy spectra given by Equations 12 and 16 for *B* ∼ 3 − 4 potentially reached in the presence of strong chorus wave‐driven electron energization and loss with or without EMIC waves.

Therefore, energy spectra *J*(*E*) of the types (Equation 12 or Equation 16) are unlikely to be produced by ULF wave‐driven inward radial diffusion alone, without additional chorus‐driven energization and loss. Some possible exceptions may be the presence of an acceleration by intense narrowband ULF waves resonating with electrons over a finite, high energy range (Degeling et al., 2008), or a peak of *J*(*E*) above 100 keV at *L* = 7–9 prior to inward radial diffusion, or an electron loss at *L* = 5–6.5 and *E* ∼ 0.3–0.6 MeV sufficiently faster than at higher energy to flatten the energy spectrum during inward radial diffusion. However, the last possibility would require electron lifetimes shorter than the inverse of the radial diffusion rate 1/*D*_*LL*_ < 0.65 day during active periods with *Kp* > 4 (Ozeke et al., 2014), which can be produced only by chorus wave‐driven quasilinear pitch‐angle diffusion toward the loss‐cone (O. V. Agapitov et al., 2019; Aryan et al., 2020) or by electron nonlinear interactions with intense chorus wave packets leading to microbursts (Miyoshi et al., 2020; X.‐J. Zhang, Angelopoulos, et al., 2022), implying a simultaneous presence of strong electron energization by the same chorus waves.

Nevertheless, during moderately disturbed periods with 100 < *AE* < 300 nT and 1 < *Kp* < 3, electron radial diffusion by ULF waves and losses due to quasi‐linear pitch angle scattering by chorus waves often become dominant at *L* = 5–7, while electron energization by chorus waves can be neglected (O. V. Agapitov et al., 2018, 2019). Although this situation does not produce extreme electron fluxes, we show in Appendix A that in this case, analytical expressions of electron lifetime *τ*_*L*_ and radial diffusion rate *D*_*LL*_ can also be combined to derive a steady‐state solution for the radial electron distribution, demonstrating that a non‐growing peak of electron PSD could be formed at lower *L* even without significant local chorus wave‐driven energization.

## 5 Comparisons Between Steady‐State Solutions and the Observed Dynamics of the Outer Radiation Belt

As noted in Section 2, the existence of steady‐state solutions to the Fokker‐Planck Equation 1 is expected to have important consequences for the outer radiation belt dynamics. Such steady‐state electron distributions should indeed represent attractors for the system dynamics, which can potentially be reached during sufficiently prolonged and active periods, provided that local chorus wave‐driven energization plays a dominant role in the evolution of the electron distribution at high energy *E* > 0.3–0.5 MeV. In this section, we analyze electron flux measurements during four different events of this kind and compare observations with both steady‐state solutions and numerical simulations.

In the past, various comparisons have been performed between electron flux variations observed during big geomagnetic storms and Fokker‐Planck simulations based on the reduced one‐dimensional Equation 1 or the full three‐dimensional diffusion equation (e.g., see Bakhareva & Orlova, 2009; Summers & Stone, 2022; Summers et al., 2002; Thorne et al., 2013). Here, we especially select long and moderate geomagnetic storms with a minimum *Dst* ∈ [−65, −49] nT, usually produced by corotating interaction regions and the associated high speed solar wind streams (Borovsky & Denton, 2006). An investigation of 19 storms within the same minimum *Dst* range in 2013–2017 has shown that 84% did produce a growing peak of ∼1.5 MeV electron PSD centered at an adiabatically invariant shell L* ∼ 4.8 ± 0.2, a characteristic of local chorus‐driven electron acceleration (Boyd et al., 2018). Selecting storms with a moderate minimum *Dst* should ensure that the *Dst* effect on electron fluxes (Kim & Chan, 1997) does not prevail over chorus‐driven energization at L* ≤ 5. Therefore, we analyze below electron fluxes measured at *L* ∼ 4.2–5, where chorus wave‐driven energization is likely dominant during these events.

Among such moderate storms, we further select events corresponding to particularly high time‐integrated activity, defined by high time‐integrated auroral electrojet index *AE* and mid‐latitude *ap* and *aa*_*H*_ range indices (for a description of these indices, see Lockwood et al., 2018; Mayaud, 1980), denoted *Int*(*AE*), *Int*(*ap*), and *Int*(*aa*_*H*_), respectively. *Int*(*AE*) and *Int*(*ap*) are integrated over continuous time intervals during which *ap* ≥ 15 nT, corresponding to *Kp* ≥ 3 (Mourenas et al., 2019). *Int*(*aa*_*H*_) is integrated over intervals where *aa*_*H*_ ≥ 18 nT (Mourenas, Agapitov, et al., 2022). During such intervals, chorus wave‐driven electron acceleration prevails over electron loss above ∼0.3–0.5 MeV (O. V. Agapitov et al., 2018, 2019; Horne et al., 2005; Mourenas, Artemyev, Agapitov, & Krasnoselskikh, 2014; Summers et al., 2002). The most active periods with *Int*(*ap*) > 2,000 nT⋅hr, *Int*(*AE*) > 20,000 nT⋅hr, or *Int*(*aa*_*H*_) > 3,000 nT⋅hr, are followed by the highest levels of 10‐day‐integrated 2‐MeV electron flux near *L* = 4.5 in the heart of the outer radiation belt (Mourenas et al., 2019; Mourenas, Agapitov, et al., 2022). Large storms produced by coronal mass ejections are usually less efficient than such moderate storms with high *Int*(*AE*) and *Int*(*ap*), caused by corotating interaction regions and high‐speed solar wind streams, in giving rise to high peaks of 2‐MeV electron flux (Miyoshi & Kataoka, 2011; Mourenas, Agapitov, et al., 2022; Spasojevic, 2014).

The 9–17 November 2003 event is the third strongest event in 1966–2020 in terms of time‐integrated geomagnetic activity *Int*(*AE*) = 135, 000 nT⋅hr (with similarly extreme *Int*(*ap*) = 9,100 nT⋅hr and *Int*(*aa*_*H*_) = 13,000 nT⋅hr), close to the highest *Int*(*AE*) levels reached in February and April 1994 (Mourenas et al., 2019). This extreme event is a one in 19‐year event in terms of time‐integrated geomagnetic activity *Int*(*AE*) (Mourenas et al., 2019), and a one in 13‐year event in terms of *Int*(*aa*_*H*_) (Mourenas, Agapitov, et al., 2022). It corresponds to a moderate geomagnetic storm, with a minimum storm time index *Dst* of −62 nT on 11 November. During the main phase of this storm, an important and prolonged solar wind dynamic pressure impulse, reaching *Pdyn* ∼ 9 nPa near midnight on 10 November, probably led to some electron loss (Boynton et al., 2017; Turner et al., 2013), explaining the low measured fluxes until 11 November. Accordingly, we analyze electron fluxes between 11 November and early 20 November, corresponding to a time‐averaged *AE* ∼ 540 nT and a time‐averaged *Kp* ∼ 4.4.

We examine electron flux variations observed by Global Positioning System (GPS) spacecraft at *L* = 4.2 and *L* = 5, close to the equator. GPS satellites have near‐circular orbits at 20,200 km, with a period of 12 hr and an inclination of 55°. Their Combined X‐ray dosimeter, developed at Los Alamos National Laboratory, measures electron fluxes in 11 energy channels between ∼0.1 and ∼6 MeV. The final fluxes are recalculated after subtraction of proton counts, using a sophisticated fitting procedure that allows to fit both decreasing and peaked electron energy spectra (Morley et al., 2016). Empirical plasmapause models as a function of *Kp* and 1‐min *AE* (O’Brien & Moldwin, 2003) place the plasmapause at *L* < 3.2–3.6 from 11 November (6 UT) to 19 November (6 UT), and later at *L* < 3.8 until early 20 November, implying that electron fluxes at *L* = 4.2–5.0 are located outside the plasmasphere during this event.

**Figure 4:**
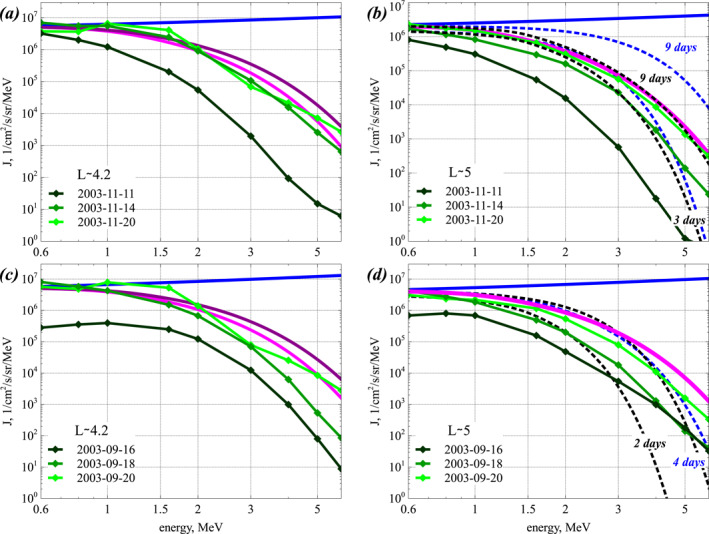
(a) Electron fluxes *J*(*E*, *t*) measured by GPS spacecraft at *L* ≃ 4.2 during the 11–19 November 2003 event (dark green to light green), with steady‐state solutions given by Equation 12 for *b* = 0 with chorus waves but without EMIC waves (blue) and given by Equation 16 with both chorus and EMIC waves for *B* = 4 (purple) and *B* = 3 (violet), using *ϵ* = 1 based on Equation 14 for the average *AE* ∼ 540 nT during this event. (b) Same as (a) for *L* = 5. Results from simulations of the full Fokker‐Planck Equation 1 are also displayed (starting from a Dirac at *E*_0_ = 0.6 MeV and *t* = 0), without EMIC waves (dashed blue), and with EMIC waves (dashed black) using *ϵ* multiplied by 4(*E* + 1/2) (*E*(*E* + 1))^3/4^ above 1.5 MeV for *B* = 4. In simulations, *J*(*E*_0_) is scaled to the measured *J*(*E*_0_) after 3 and 9 days. Typical wave and plasma parameters for average *Kp* ∼ 4–5 and *AE* ∼ 500–600 nT are used, corresponding to *D*_*EE*_/*E* ^2^ ∼ 0.15 day^−1^. (c and d) Same as (a and b) for the 16–20 September 2003 event, with *ϵ* = 0.9 based on Equation 14 for an average *AE* ∼ 600 nT during this event.

Figures 4a and 4b show the evolution of the electron flux *J*(*E*, *t*) measured by GPS spacecraft at *L* ≃ 4.2 and *L* ≃ 5 during this extreme event (green curves). Note that *Dst* varies by less than 30 nT between the different times at which the measured flux is displayed, corresponding to less than 12.5% (7.5%) of the equatorial background magnetic field strength at *L* = 5 (*L* = 4.2). This should produce only a weak *Dst*‐effect on electron fluxes (Kim & Chan, 1997), negligible to first order compared with chorus wave‐driven electron energization and loss. The maximum energy of injections was estimated as ∼350–450 keV based on a wider range of temporary flux variations at lower energy. Accordingly, we use *E*_0_ = 600 keV for the minimum energy where the flux is assumed to remain constant in analytical steady‐state solutions and simulations.

Figures 4a and 4b also show the analytical steady‐state *J*(*E*) solution given by Equation 12 for *b* = 0 in the presence of chorus waves but without EMIC waves (solid blue), and the steady‐state solution given by Equation 16 for *B* = 4 in the presence of both chorus and EMIC waves (purple). It is worth noting that the steady‐state solution given by Equation 12 only depends on the parameter *ϵ*, whereas the steady‐state solution given by Equation 16 depends on both *ϵ* and *B* parameters. Since sufficiently comprehensive (in time, MLT, and latitude) wave and plasma measurements are not available during this event (as well as during the following events in this section), we use the statistical estimate of *ϵ* given by Equation 14 based on the average *AE* during the event (this gives here *ϵ* ≃ 1 for an average *AE* ∼ 540 nT). The empirical Equation 14 has been derived from 2012 to 2017 Van Allen Probes statistics of bounce‐ and MLT‐averaged chorus‐driven energy and pitch‐angle diffusion rates (O. V. Agapitov et al., 2019). In the presence of both EMIC and chorus waves, we also use in the steady‐state solution given by Equation 16 a statistical estimate of *B*, *B* ∼ 3–4 for *Kp* > 4 and *AE* > 400 nT based on spacecraft statistics of EMIC and chorus waves at *L* ∼ 5 (see Section 3).

The results of numerical simulations solving the full Fokker‐Planck Equation 1 are displayed in Figure 4b at *L* = 5, with EMIC waves for *B* = 4 (dashed black) and without EMIC waves (dashed blue). In these simulations, we use the same *ϵ* and *B* parameters as above, together with the statistical chorus wave power and *f*_*pe*,*acc*_/*f*_*ce*_ parameters from O. V. Agapitov et al. (2018, 2019) based on the average *AE* during the event. The corresponding typical chorus wave and plasma parameters at *L* = 5 for an average *AE* ∼ 500–600 nT and an average *Kp* ∼ 4–5, as during this event, are a time‐ and MLT‐averaged *B*_*w*,*acc*_ ≃ 55 pT, *f*_*m*_/*f*_*ce*_ = 0.25, Δ*θ* = 30°, and *f*_*pe*,*acc*_/*f*_*ce*_ = 4, giving *D*_*EE*_/*E* ^2^ ≃ 0.15 day^−1^ at 1 MeV (O. V. Agapitov et al., 2018, 2019; Mourenas, Artemyev, Agapitov, & Krasnoselskikh, 2014). The corresponding effective lifetimes of >1.5 MeV electrons in the presence of chorus and EMIC waves are $\tau_{L}^{\mathrm{eff}}≃1.6$ days, consistent with the minimum lifetimes of ∼1.5–5 MeV electrons measured by ELFIN CubeSats at *L* ∼ 5.0–6.5 outside the plasmasphere (Mourenas et al., 2021) and by the Van Allen Probes at *L* = 5.5–6.0 probably outside the plasmasphere (Claudepierre et al., 2020). As noted in Section 3, such a prolonged intense event with *ϵ* ≤ 1 should allow us to assess the presence or not of EMIC waves in high‐density regions in the dusk sector together with chorus waves in the night/dawn sector of lower density.

At both *L* = 4.2 and *L* = 5, the hardest electron energy spectra *J*(*E*) measured on early 20 November 2003 agree well with the steady‐state solution given by Equation 16 for *B* = 4 in the presence of both chorus and EMIC waves (purple). The increase of the measured electron flux strongly slows down as it approaches this steady‐state solution at *L* = 4.2 and *L* = 5 in Figures 4a and 4b, in good agreement with numerical simulations including both chorus and EMIC waves (dashed black) in Figure 4b. Additional simulations performed in Figure 4b show that, without EMIC waves, this event should have led to a higher plateau of *J*(*E*) extending up to ∼3 MeV after 9 days (dashed blue), whereas the observed plateau ends at ∼1.5 MeV as in simulations with EMIC waves (dashed black). Without EMIC waves, the slight reduction of average geomagnetic activity during the 14‐20 November period, which corresponds to 6 days of average *Kp* = 4.2 (among which 5 days of average *Kp* = 4.5) compared to only 3 days of average *Kp* = 4.7 on 11–14 November, cannot account for the modest flux increase observed at both *L* = 4.2 and *L* = 5 between 14 and 20 November after a large increase between 11 and 14 November (O. V. Agapitov et al., 2018, 2019). Therefore, these results provide evidence of the strong impact of EMIC waves on lifetimes of >1.5 MeV electrons at *L* ≃ 4.2–5.0, leading to similar energy spectra after ∼3 days and ∼9 days of sustained electron energization and loss, close to the analytical steady‐state solution given by Equation 16 with *B* = 4.

Interestingly, at *L* = 4.2 the shape of the electron energy spectrum on 20 November is closer to the steady‐state solution given by Equation 12 without EMIC waves (solid blue) than to the solution given by Equation 16 with EMIC waves (purple) below 1.6 MeV, contrary to observations at *L* = 5. Since the minimum electron energy E* for cyclotron resonance with EMIC waves is proportional to the local *f*_*ce*_/*f*_*pe*_ (Mourenas et al., 2016; Summers & Thorne, 2003) in a duskside plasmaspheric plume or plasmasphere boundary region where empirical statistics give a scaling *f*_*ce*_/*f*_*pe*_ ≈ 1/*L* (Ozhogin et al., 2012; Sheeley et al., 2001), this could be due to a slightly higher time‐averaged E* ≃ 2 MeV at lower *L*, in agreement with EMIC wave statistics for *Pdyn* > 2 nPa as during this event (Ross et al., 2021).

The second investigated period, on 16–20 September 2003, corresponds to an event similar to, although twice shorter than, the November 2003 event, with *Int*(*AE*) = 74, 000 nT⋅hr (and *Int*(*ap*) = 5, 200 nT⋅hr, *Int*(*aa*_*H*_) = 7, 160 nT⋅hr) and time‐averaged *AE* ∼ 600 nT, corresponding to *ϵ* ≃ 0.9 based on Equation 14, and *Kp* ∼ 4.5. This event is accompanied by a moderate storm with a minimum *Dst* = −65 nT and a *Dst* variation smaller than 20 nT between the times at which the measured electron fluxes are displayed in Figures 4c and 4d. Based on empirical plasmapause models (O’Brien & Moldwin, 2003), the plasmapause remains at *L* < 3.7 during this whole event. At the end of this event, on 20 September 2003, the measured energy spectrum is intermediate between energy spectra measured on 14 and 20 November 2003. Clearly, ∼2–3 additional days of similar chorus wave‐driven energization would have been needed after 20 September for *J*(*E*) to reach the approximate steady‐state solution given by Equation 16 for *B* = 4 above 2.5 MeV. Figure 4d further shows that during such events lasting less than 4 days, the fluxes *J*(*E*, *t*) obtained by numerically solving the Fokker‐Planck Equation 1 can remain roughly similar with (dashed black) and without (dashed blue) EMIC waves, until *J*(*E*, *t*) without EMIC waves increases beyond the level of the steady‐state solution with EMIC waves.

At *L* = 4.2, the electron energy spectrum on 20 September is again closer to the steady‐state solution given by Equation 12 without EMIC waves (solid blue) below 1.6 MeV than to the solutions given by Equation 16 with EMIC waves (purple and violet). Therefore, Figures 4a and 4c demonstrate that the steady‐state solution given by Equation 12 without EMIC waves can be reached over 0.6–1.6 MeV at *L* ≃ 4.2 after ∼4–9 days of strong and sustained chorus wave‐driven electron energization. This suggests that the average minimum electron energy E* for cyclotron resonance with EMIC waves could be systematically slightly higher than 1.6 MeV at *L* ≤ 4.2, in agreement with previous studies based on measured electron lifetimes and EMIC wave statistics (Mourenas et al., 2017, 2021; Ross et al., 2021). In this case, the steady‐state solution is approximately given by Equation 16 with $\alpha =\max\left(1/2,\tanh\left({\left[1.5E/E^{*}\right]}^{2}\right)\right)$.

Next, we examine electron flux variations measured by the Van Allen Probes (Claudepierre et al., 2021; Mauk et al., 2013) near the equator at adiabatically invariant shells L* = 4 and L* = 5 (determined using the TS04 magnetic field model, see Tsyganenko & Sitnov, 2005) on 21–25 April 2017. This period corresponds to another strong, but less important and shorter, time‐integrated geomagnetic event reaching *Int*(*AE*) = 40,500 nT⋅hr (and *Int*(*ap*) = 2,600 nT⋅hr, *Int*(*aa*_*H*_) = 4,900 nT⋅hr). The average *AE* was ∼530 nT from 13 UT on 21 April to 13 UT on 25 April (corresponding to *ϵ* ≃ 1 based on Equation 14), with an average *Kp* ≃ 4 and only a moderate minimum *Dst* = −51 nT (and less than 20 nT variation of *Dst* between times at which fluxes are displayed). This event, one of the most important in 2013–2017 in terms of *Int*(*AE*) and *Int*(*aa*_*H*_), produced the highest 10‐day‐integrated 2‐MeV electron flux at L* ∼ 4.5 recorded during that period (Mourenas, Agapitov, et al., 2022). Based on empirical plasmapause models (O’Brien & Moldwin, 2003), the plasmapause remained at *L* < 3.8 between 21 and 25 April. We use daily averaged level‐2 spin‐averaged omnidirectional electron fluxes measured by the Magnetic Electron Ion Spectrometer (MagEIS, see Claudepierre et al., 2021) at *E* ≤ 3.6 MeV. MagEIS forms part of the Energetic Particle, Composition, and Thermal Plasma (ECT) Suite (Spence et al., 2013). Analytical steady‐state solutions and fluxes in simulations are normalized to measured flux at *E*_0_ = 500 keV, because variations in measured flux increase at lower energy during the last 3 days at both L* = 4 and L* = 5.

**Figure 5:**
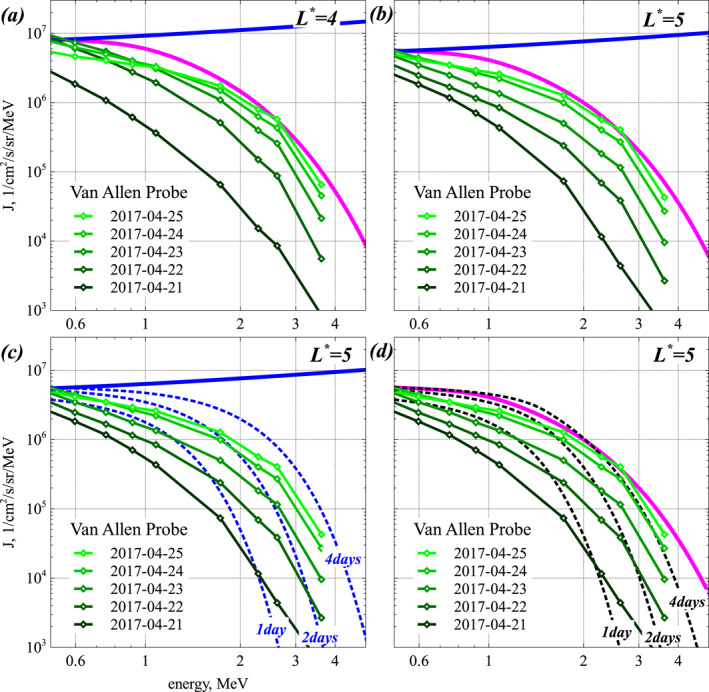
Comparisons between electron fluxes *J*(*E*, *t*) measured near the magnetic equator by the Van Allen Probes (dark green to light green) during the 21–25 April 2017 event and steady‐state solutions given by Equation 12 for *b* = 0 with intense chorus waves but without EMIC waves (solid blue), and given by Equation 16 for *B* = 4 (purple) with intense chorus and EMIC waves. (a) L* ≃ 4.0. (b) L* = 5.0. (c) Same as (b) together with numerical simulations based on the full Fokker‐Planck Equation 1 with chorus but without EMIC waves, using the same wave and plasma parameters as in Figure 4 with *ϵ* = 1, typical for *Kp* ∼ 4–5 and *AE* ∼ 500–600 nT (dashed blue curves). In simulations, *J*(*E*_0_ = 0.5 MeV) is normalized to the measured *J*(*E*_0_) on the corresponding day. (d) Same as (c) but showing numerical simulations based on the full Fokker‐Planck Equation 1 with chorus and EMIC waves, where *ϵ* is multiplied by 4(*E* + 1/2) (*E*(*E* + 1))^3/4^ at *E* > 1.5 MeV for *B* = 4 (dashed black curves).

In Figures 5a and 5b at both L* = 4 and L* = 5, the measured electron fluxes *J*(*E*, *t*) at the end of this event on 25 April 2017 (green curves) approach the steady‐state solution given by Equation 16 for *B* = 4 and *ϵ* = 1 in the presence of both chorus and EMIC waves (purple curve). The increase of measured fluxes at 2.0–3.6 MeV is much slower from 23 to 25 April at L* = 4 and L* = 5, despite a similar average *AE* as from 21 to 23 April, suggesting an approach to a stationary state. Numerical simulations including both chorus and EMIC waves in Figure 5d —using the same parameters as for the previous events—demonstrate a strong deceleration of the increase of 2.0–3.6 MeV electron fluxes (dashed black curves) as they reach the corresponding steady‐state solution (purple), in rough agreement with observations. In contrast, Figure 5c shows a large discrepancy, after 4 days, between measured fluxes at ∼2.3–3.0 MeV and the higher fluxes from simulations assuming an absence of EMIC waves (dashed blue curves), although such simulated fluxes correspond to observations on 23 April after 2 days. Without additional electron loss provided by EMIC waves, chorus wave‐driven electron acceleration could indeed increase fluxes over the long term up to the much higher stationary solution given by Equation 12 with *b* = 0 (solid blue curve), which corresponds to a balance between chorus‐driven electron acceleration and loss and yields a nearly flat *J*(*E*) above 0.5 MeV (various examples of full 3D simulations showing this long‐term behavior have been provided by Hua et al. [2022]).

**Figure 6:**
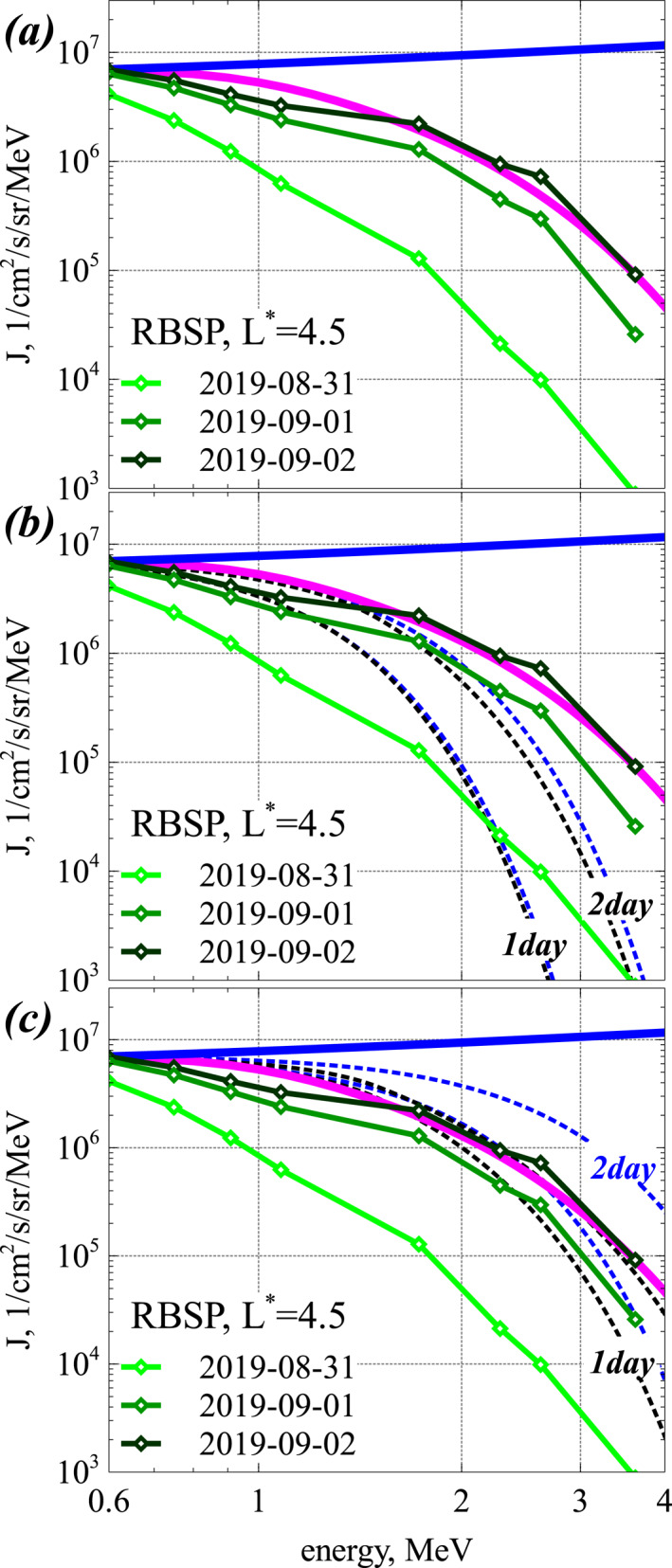
(a) Comparisons between electron fluxes *J*(*E*, *t*) measured by the Van Allen Probes at L* ∼ 4.5 during the 31 August to 2 September 2019 event (light green to dark green) and steady‐state solutions given by Equation 12 for *b* = 0 with chorus but without EMIC waves (solid blue) and given by Equation 16 for *B* = 4 with chorus and EMIC waves (purple), using *ϵ* = 1 and the same other wave and plasma parameters as in Figure 4. (b) Same as (a), together with numerical simulation results after 1 and 2 days, based on the full Fokker‐Planck Equation 1 for *ϵ* = 1 and *D*_*EE*_/*E* ^2^ = 0.15 day^−1^ at 1 MeV, with either only chorus waves (dashed blue curves), or both chorus and EMIC waves with *ϵ* multiplied by 4(*E* + 1/2) (*E*(*E* + 1))^3/4^ at *E* > 1.5 MeV for *B* = 4 (dashed black curves). (c) Same as (b) but using *D*_*EE*_/*E* ^2^ = 0.45 day^−1^ at 1 MeV.

Between January 2019 and May 2022, the strongest time‐integrated geomagnetic event, with *Int*(*ap*) = 2,340 nT⋅hr, occurred from 18 UT on 30 August to 09 UT on 2 September 2019, also reaching *Int*(*aa*) ≃ 4,400 nT⋅hr from 12 UT on 30 August to 24 UT on 2 September. It was accompanied by a moderate storm with a minimum *Dst* of −52 nT at 6 UT on 1 September. We examine the period between 31 August and 2 September 2019, which corresponds to an average *Kp* ≃ 4.5. The corresponding *AE* index is not yet available, but since the average *Kp* is similar as during the November 2003 and April 2017 events, we use *ϵ* ≃ 1 as for these events. Based on empirical plasmapause models (O’Brien & Moldwin, 2003), the plasmapause remained at *L* < 4 during this event, and *Dst* varied by less than 20 nT between the times at which electron fluxes measured at L* = 4.5 are displayed in Figure 6. During this event, a growing peak of PSD of ∼2–4 MeV electrons has been observed by the Van Allen Probes at L* = 4–5, which was not reproduced by simulations including ULF wave‐driven electron inward radial diffusion without local chorus‐driven acceleration, suggesting an important effect of chorus‐driven electron acceleration (Hudson et al., 2021).

Figure 6a shows comparisons between steady‐state solutions and daily averaged electron fluxes *J*(*E*, *t*) measured during this event at L* ∼ 4.5 by the Van Allen Probes. As during the three preceding events, the measured fluxes on 2 September nearly reach the steady‐state solution given by Equation 16 for *B* = 4 corresponding to a presence of both chorus and EMIC waves (purple curve). As during the November 2003 and April 2017 events, the flux increase is much slower from 1 to 2 September than from 31 August to 1 September, despite similar average *Kp* values (*Kp* = 4.3 vs. *Kp* = 4.7), consistent with an approach to a stationary state. However, the steady‐state solution is attained here after only 2 days, whereas it was reached after at least ∼4–5 days during the three preceding events. On 1 September 2019, after only one day of sustained substorm activity, *J*(*E*, *t*) has already very strongly increased compared to its level on 31 August, reaching a normalized level *J*(*E*)/*J*(*E*_0_) at 3 MeV comparable to the level reached on 14 November 2003 after 3 days of sustained chorus wave‐driven energization. This suggests the presence of a much stronger energization during the September 2019 event. Figure 6b indeed shows that using in simulations the same chorus‐driven energy diffusion rate *D*_*EE*_/*E* ^2^ = 0.15 day^−1^ and *ϵ* ∼ 1 at 1 MeV as during the three preceding events clearly does not allow to recover observations, with or without EMIC waves. In contrast, simulations with a three times larger *D*_*EE*_/*E* ^2^ = 0.45 day^−1^ at 1 MeV relatively well reproduce observations in Figure 6c after 1 day, both with and without EMIC waves, and after 2 days only with EMIC waves.

Based on High Frequency Receiver (HFR) measurements of the upper hybrid resonance frequency onboard the Van Allen Probes (Kurth et al., 2015) at L* ∼ 4.5 and 9–10 MLT during this event, the MLT‐averaged electron plasma frequency to gyrofrequency ratio in the night/dawn sector could have been *f*_*pe*,*acc*_/*f*_*ce*_ ∼ 3.5, slightly smaller than the value *f*_*pe*,*acc*_/*f*_*ce*_ ∼ 4 used in Figure 6b. Assuming such a smaller plasma density, the higher energy diffusion rate during this event could correspond to a ∼2 times higher MLT‐averaged chorus wave power at all latitudes (with *B*_*w*,*acc*_ ∼ 75 pT) than its typical level for *Kp* ∼ 4.5. This high time‐averaged chorus wave power suggests the probable presence of nonlinear interactions between relativistic electrons and intense chorus wave packets during this event (Albert et al., 2013; Demekhov et al., 2006; Katoh et al., 2008; Kubota & Omura, 2018; Mourenas, Zhang, et al., 2022; X. J. Zhang, Agapitov, et al., 2020). Such nonlinear interactions can increase the effective scattering rates of electrons by factors of ∼1.5–2, over periods of hours to days, compared with quasi‐linear diffusion by waves of moderate time‐averaged amplitudes (Artemyev et al., 2021, 2022). Accordingly, nonlinear interactions could have provided part of the required increase of the effective electron diffusion rates during this event.

**Figure 7:**
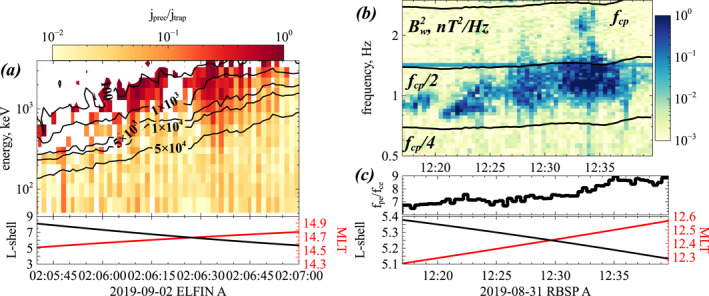
(a) Time interval of high precipitating‐to‐trapped electron flux ratio *j*_*prec*_/*j*_*trap*_, peaking at ∼1.5–3 MeV, measured by ELFIN A CubeSat on 2 September 2019 at *L* ∼ 5–7 near 15 MLT, typical of EMIC wave‐driven precipitation. Black contours show magnitude of trapped fluxes, *j*_*trap*_, in units of 1/cm^3^/s/sr/keV. The lower part shows spacecraft *L* (black) and MLT (red) locations. (b) Hydrogen band EMIC waves observed by the Van Allen Probes at *L* ≃ 5 on 31 August 2019 around 12:35 UT during the same event. (c) *f*_*pe*_/*f*_*ce*_ ratio during Van Allen Probes observations in (b). The lower part shows spacecraft *L* (black) and MLT (red) locations.

Finally, Figure 7a shows a time interval of high precipitating‐to‐trapped electron flux ratio *j*_*prec*_/*j*_*trap*_ measured at *L* ∼ 5–7 in the dusk sector (near 15 MLT) by the ELFIN A CubeSat (Angelopoulos et al., 2020) at low altitude during the same September 2019 event. The precipitating‐to‐trapped electron flux ratio steeply increases above 1 MeV, reaching a peak level *j*_*prec*_/*j*_*trap*_ ∼ 0.5–1 at ∼1.5–3 MeV, a characteristic feature of EMIC wave‐driven electron precipitation (Grach et al., 2022). Figure 7b shows intense hydrogen band EMIC waves recorded during the same event by the Electric and Magnetic Field Instrument Suite and Integrated Science (EMFISIS) Instrument Suite (Kletzing et al., 2013) on board the Van Allen Probes, near 13 MLT at *L* ∼ 5.1, corresponding to L* ∼ 4.7. The EMFISIS Instrument Suite consists of a tri‐axial fluxgate magnetometer, a tri‐axial search coil magnetometer, and a sweep frequency receiver. The corresponding plasma frequency to electron gyrofrequency ratio *f*_*pe*_/*f*_*ce*_ ≃ 8–9 inferred from the upper hybrid resonance line (Kurth et al., 2015) is displayed in Figure 7c. Such hydrogen band EMIC waves have a main peak of wave power extending up to a frequency *f* ∼ 0.53 *f*_*cp*_ (with *f*_*cp*_ the proton gyrofrequency), together with a second peak of wave power at *f* ∼ 0.73 *f*_*cp*_. Based on the absence of intense hydrogen band EMIC waves at *f* < 0.32 *f*_*cp*_ in Figure 7b after 12:25 UT, it is reasonable to assume that the ion composition consists of more than 94% protons (Kersten et al., 2014). This gives a minimum electron energy *E*_min_ ∼ 1.5 MeV for cyclotron resonance with waves at *f* ∼ 0.73 *f*_*cp*_, and *E*_min_ ∼ 2.8 MeV for *f* ∼ 0.53 *f*_*cp*_ (Mourenas et al., 2016; Summers & Thorne, 2003). These observations therefore confirm the presence of EMIC wave‐driven electron precipitation above ∼1.5 MeV near L* ∼ 4.5 on the dusk side during this September 2019 event.

## 6 Conclusions

In this paper, we have provided realistic analytical steady‐state solutions to the Fokker‐Planck diffusion equation describing chorus wave‐driven energization and loss of 0.5–5 MeV electrons in the outer radiation belt in the quasi‐linear regime, in the presence of sustained low‐energy (∼1–300 keV) electron injections from the plasma sheet. Comparisons with full numerical solutions have shown that such steady‐state electron distributions represent attractors for the system dynamics. Therefore, such steady‐state solutions should correspond to the hardest electron energy spectra potentially encountered in the outer belt during prolonged periods of sustained low‐energy electron injections.

The dependencies of these steady‐state solutions on wave and plasma parameters, as well as geomagnetic activity, have been provided based on empirical statistical models of chorus waves and plasma density. The crucial parameters are chorus wave power latitudinal distribution and background plasma density in the night/morning sector, and the strength of electron injections. Such steady‐state solutions can be reached only after sufficiently strong and prolonged injections of low‐energy electrons, providing both chorus wave growth and seed electrons subsequently accelerated by chorus waves. This should correspond to the periods of highest time‐integrated geomagnetic activity, as measured by integrated *AE*, *ap*, *aa*, or *aa*_*H*_ indices above some threshold corresponding to a prevalence of electron acceleration over loss. Steady‐state solutions in the simultaneous presence of realistic chorus and EMIC waves in different MLT sectors have also been provided, showing that the resulting much faster electron loss above ∼1.5 MeV strongly modifies the steady‐state electron energy spectrum at high energy, making it much less hard (i.e., more steeply decreasing at higher energy) than without EMIC waves.

Comparisons with GPS electron flux measurements during an event of extreme time‐integrated geomagnetic activity in November 2003 have shown the likely presence of both chorus and EMIC waves (in different MLT sectors) and their strong combined impact on lifetimes of *E* > 1.5 MeV electrons. At the end of this event, the electron flux *J*(*E*, *t*) reached the analytical steady‐state solution given by Equation 12 without EMIC waves up to ∼1.6 MeV at *L* = 4.2, after ∼4–9 days of sustained chorus wave‐driven energization. But at both *L* = 4.2 and *L* = 5, *J*(*E*, *t*) was found to be strongly limited by combined EMIC and chorus wave‐driven electron precipitation above 1.5 MeV, reaching the corresponding approximate analytical steady‐state solution given by Equation 16, with much lower fluxes than the solution given by Equation 12, after ∼5 − 9 days of sustained chorus wave‐driven energization. A similar behavior was found during strong events in April 2017 and September 2019 analyzed using Van Allen Probes data (see also Hua et al., 2022). Although statistical values of wave and plasma parameters for a given average *AE* have been used for the comparisons between analytical solutions and electron flux observations, the similarly good agreement during the November 2003 and April 2017 events, with similar average *AE*, suggests that these statistical values are reliable over sufficiently long time periods of 4–9 days.

During these three events, the increase of *J*(*E*, *t*) slowed down as it approached the steady‐state solution given by Equation 16, and *J*(*E*, *t*) nearly displayed the same asymptotic, steady‐state energy spectrum shape after only ∼2 to ∼4 days. However, the impact of EMIC waves on *J*(*E*, *t*) can usually be identified only after at least ∼4 days of sustained substorm activity, unless chorus‐driven energy diffusion is exceptionally strong. At earlier times, *J*(*E*, *t*) remains similar with and without EMIC waves. These results demonstrate that the approximate analytical steady‐state solution given by Equation 16 with *B* ∼ 3–4 provides a realistic estimate of the most extreme electron energy spectrum and can be reliably used to estimate the worst case risk of total ionizing dose for satellite electronics, in combination with previous predictive models of periods of high and prolonged ∼2 MeV electron flux (Mourenas et al., 2019; Mourenas, Agapitov, et al., 2022).

During the September 2019 event, a noticeably faster increase of 2–4 MeV electron fluxes was observed, suggesting the presence of stronger chorus wave power, smaller plasma density, and/or stronger nonlinear wave‐particle interactions potentially speeding up the approach to the steady state (Artemyev et al., 2021, 2022). Note, however, that the steady‐state solution could be slightly modified by strong nonlinear interactions over some short time intervals, because this steady state depends on the factor *ϵ* ∼ *D*_*αα*_/*D*_*EE*_, and the energy diffusion rate *D*_*EE*_ of high equatorial pitch‐angle ∼1 MeV electrons can be more easily increased by nonlinear interactions than the pitch‐angle diffusion rate *D*_*αα*_ of ∼1 MeV electrons around the loss‐cone, due to the higher latitude of cyclotron resonance with low to medium pitch‐angle electrons (Tao & Bortnik, 2010) and the lower chorus wave power there (O. V. Agapitov et al., 2018). Accordingly, further work will be needed to carefully assess the importance of nonlinear interactions over the course of storms.

In addition, we briefly examined steady‐state solutions for the radial electron distribution in the presence of both radial diffusion by ULF waves and electron precipitation by chorus waves, but with negligible chorus wave‐driven electron acceleration, during periods of moderate geomagnetic activity. In this case, the analytical steady‐state radial electron phase space density decreases fast toward higher *L* for fixed first adiabatic invariant corresponding to *E* = 1–4 MeV at *L* = 5. Since such steady states also represent attractors for the system dynamics, their existence could favor the appearance of these particular gradients in the outer radiation belt during moderate disturbances.

## Data Availability

Van Allen Probes MagEIS electron flux data (REL03 L2) is available at https://rbsp-ect.newmexicoconsortium.org/data_pub/rbspa/mageis, while EMFISIS data is available from https://emfisis.physics.uiowa.edu/data/index. LANL CXD data of GPS electron flux is available from NOAA at https://www.ngdc.noaa.gov/stp/space-weather/satellite-data/satellite-systems/gps/. ELFIN data is available at https://data.elfin.ucla.edu/. The *aa*_*H*_ index can be retrieved at https://www.swsc-journal.org/articles/swsc/olm/2018/01/swsc180022/swsc180022-2-olm.txt. OMNI data of *AE*, *ap*, *Dst*, *Kp*, and *Pdyn* are available from the GSFC/SPDF OMNIWeb interface at https://omniweb.gsfc.nasa.gov. The *aa* index is available from ISGI, Ecole et Observatoire des Sciences de la Terre, at http://isgi.unistra.fr.

## Appendix A Analytical Steady‐State Radial Electron Distribution Due To Radial Diffusion and Chorus‐Driven Loss

Let us briefly examine the case where electron radial diffusion by ULF waves and loss due to quasi‐linear pitch angle scattering by chorus waves are dominant, and electron energization by chorus waves is negligible. At *L* = 5–7, this corresponds to 100 < *AE* < 300 nT and 1 < *Kp* < 3 (O. V. Agapitov et al., 2018, 2019). We focus on nearly equatorially mirroring electrons, representing the majority of the trapped population. For dominant electrostatic ULF perturbations in the radial diffusion rate, *D*_*LL*_ is weakly dependent on pitch angle *α* (Ozeke et al., 2014). Electron lifetimes *τ*_*L*_ due to chorus‐driven pitch angle scattering are also weakly dependent on *L* (Aryan et al., 2020). Radial diffusion and pitch angle scattering by chorus waves can then be considered as approximately independent, the variable *ζ* = *μ*/sin^2^ *α* = *p* ^2^/*B* (with *B* the geomagnetic field strength) is nearly conserved by both pitch angle and radial diffusion operators, and the evolution of the electron PSD *f* at fixed *ζ* can be described by a simplified Fokker‐Planck equation (Schulz & Lanzerotti, 1974):

(A1)
$$\frac{\partial f}{\partial t}=L^{5/2}\frac{\partial }{\partial L}\left(\frac{D_{LL}}{L^{5/2}}\frac{\partial f}{\partial L}\right)-\frac{f}{\tau_{L}}.$$

Based on previous statistics of ULF waves, we have approximately *D*_*LL*_ ∼ *D*_0_ *L* ^9^ day^−1^ over 5 < *L* < 8, with *D*_0_ ≃ 2.2 × 10^−9+0.461^ ^*Kp*^ (Ozeke et al., 2014). For a constant *ζ* ∼ (*E*(*E* + 1))*L* ^3^, the lifetime *τ*_*L*_ ∼ 2.8 (2*E* + 1)[*E*(*E* + 1)]^3/4^ days of *E* > 0.1 MeV electrons during periods with *AE* ∈ [100, 300] nT or *Kp* ∈ [1, 3] (O. V. Agapitov et al., 2018; Aryan et al., 2020) can be rewritten as $\tau_{L}∼{\left(L_{\min}/L\right)}^{15/4}\tau_{L}\left[E_{\max}\right]$ days, with *E*_max_ = *E*[*L*_min_]. Steady‐state solutions *f*(*L*, *ζ*) to Equation A1 must then satisfy

(A2)
$$\frac{\partial^{2}f}{\partial L^{2}}+\frac{13}{2L}\frac{\partial f}{\partial L}-\frac{f}{C\;L^{21/4}}=0$$

with $C=D_{0}L_{\min\;}^{15/4}\tau_{L}\left[E_{\max}\right]$ for *E* > 0.1 MeV. Equation A2 is an Emden‐Fowler equation (Fowler, 1931), with an always positive solution

(A3)
$$f\left(L\right)=\frac{1}{L^{11/4}}\;I_{\frac{22}{13}}\left[\frac{8\;L^{-13/8}}{13\;\sqrt{C}}\right],$$

with *I* the modified Bessel function of the first kind. Similar analytical steady‐state solutions, for a slightly different radial diffusion equation with a lifetime varying as a power‐law of *L*, have been found in previous works (Haerendel, 1968; Thorne, 1972). For *L*_min_ = 5, this steady‐state *f*(*L*) decreases like *f*(*L*) ∼ 1/*L* ^9^ to ∼1/*L* ^6^ over *L* = 5–8 for *E*_max_ = 0.5–4.0 MeV or *ζ* = 210–8,300 MeV/G.

As the effective radial diffusion rate depends on both *D*_*LL*_ and the preexisting radial PSD gradient *∂f*/*∂L* (Schulz & Lanzerotti, 1974), the steady‐state solution given by Equation A3 corresponds to the radial PSD gradient that finely tunes the balance, at each *L*, between PSD increase due to radial diffusion and PSD decrease due to precipitation. Since electron precipitation is faster at higher *L* for constant *ζ*, steady‐state solutions with a decreasing electron PSD toward higher *L* can appear even in the case of an initial PSD peak located at higher *L*, provided that this peak does not remain fixed.

The stationary solution given by Equation A3 should represent an attractor for the system dynamics, because *f*(*L*, *ζ*, *t*) varies more slowly in its vicinity. This attractor state with a maximum of electron PSD at low *L* can be initiated by various physical processes, alone or in combination: local chorus wave‐driven energization (Horne et al., 2005), inward radial diffusion rapidly followed by outward radial diffusion toward a suddenly reduced PSD at higher *L* (Ozeke et al., 2020; Pinto et al., 2020), local interactions with intense narrowband ULF waves (Degeling et al., 2008), or a stronger precipitation at higher *L* (Green & Kivelson, 2004) as in the case of chorus wave‐driven electron loss at fixed *ζ* ≃ *μ*. The existence of this attractor state given by Equation A3 could favor observations of a peak of ∼1–4 MeV electron PSD at *L* ≃ 5 in the outer radiation belt (Boyd et al., 2018; Y. Chen et al., 2007; Turner et al., 2012) even without chorus‐driven electron energization. However, this PSD peak cannot grow in time without significant local chorus‐driven electron energization. The prevalence of chorus‐driven electron precipitation should lead to a progressive decrease of *f*(*L*) at all *L*, during which the radial shape of *f*(*L*) comes close to the shape of the steady‐state solution given by Equation A3.
